# Plant species- and stage-specific differences in microbial decay of mangrove leaf litter: the older the better?

**DOI:** 10.1007/s00442-021-04865-3

**Published:** 2021-02-09

**Authors:** Novia Arinda Pradisty, A. Aldrie Amir, Martin Zimmer

**Affiliations:** 1 Faculty 2 Biology/Chemistry, Universität Bremen, Bremen, Germany; 2 Institute for Marine Research and Observation, Ministry of Marine Affairs and Fisheries, Negara, Bali Indonesia; 3 Institute for Environment and Development (LESTARI), Universiti Kebangsaan Malaysia, Bangi, Selangor Malaysia; 4 IUCN SSC Mangrove Specialist Group, London, England; 5 Leibniz Centre for Tropical Marine Research, ZMT-GmbH, Bremen, Germany

**Keywords:** Leaf litter, Microbial decay, Leaf chemistry, Extracellular enzyme activity, Mangrove management

## Abstract

Leaf litter and its breakdown products represent an important input of organic matter and nutrients to mangrove sediments and adjacent coastal ecosystems. It is commonly assumed that old-grown stands with mature trees contribute more to the permanent sediment organic matter pool than younger stands. However, neither are interspecific differences in leaf decay rates taken into account in this assumption nor is our understanding of the underlying mechanisms or drivers of differences in leaf chemistry sufficient. This study examines the influence of different plant species and ontogenetic stage on the microbial decay of mangrove leaf litter. A litterbag experiment was conducted in the Matang Mangrove Forest Reserve, Malaysia, to monitor leaf litter mass loss, and changes in leaf litter chemistry and microbial enzyme activity. Four mangrove species of different morphologies were selected, namely the trees *Rhizophora apiculata* and *Bruguiera parviflora*, the fern *Acrostichum aureum* and the shrub *Acanthus ilicifolius*. Decay rates of mangrove leaf litter decreased from *A. ilicifolius* to *R. apiculata* to *B. parviflora* to *A. aureum.* Leaf litter mass, total phenolic content, protein precipitation capacity and phenol oxidase activity were found to decline rapidly during the early stage of decay. Leaf litter from immature plants differed from that of mature plants in total phenolic content, phenolic signature, protein precipitating capacity and protease activity. For *R. apiculata*, but not of the other species, leaf litter from immature plants decayed faster than the litter of mature plants. The findings of this study advance our understanding of the organic matter dynamics in mangrove stands of different compositions and ages and will, thus, prove useful in mangrove forest management.

**Supplementary Information:**

The online version contains supplementary material available at 10.1007/s00442-021-04865-3.

## Introduction

Mangroves are an ecological assemblage of trees, palms, shrubs and ferns adapted to grow above mean sea level in the upper intertidal region of coastal and estuarine environments in tropical and sub-tropical latitudes (Duke, 2011; Feller et al. 2010). As one of the most productive ecosystems, mangroves store vast amounts of organic matter in their sediment, where anoxic conditions result in a slow turnover of accumulated detritus from various sources (Donato et al. 2011; Murdiyarso et al. 2015). Thus, they contribute significantly to climate change-mitigation through their "blue carbon" stocks. Organic matter in mangrove sediments originates from detritus of both local sources (i.e. mangroves, microphytobenthos) and tidal inputs (e.g. phytoplankton, seagrass and macroalgae), with varying relative inputs of these sources both within and among different mangrove sites (Bouillon et al. 2004).

Apart from their important role in greenhouse gas sequestration, mangroves also provide various economic and societal benefits for human wellbeing, from securing food and non-food resources to nature-based solutions for coastal protection and recreation (van Oudenhoven et al. 2015). Wood extraction is among the oldest uses of natural resources provided by mangroves. Sustainable silvicultural management, implementing rotation cycles and selective logging practices, that allow for recolonization and regrowth of dense mangrove stands between clear-felling events has been practiced in only a few places, such as the Matang Mangrove Forest Reserve (MMFR) in Peninsular Malaysia, the Bintuni Bay Mangroves in West Papua, Indonesia, and the Sundarbans in Bangladesh (Ariffin and Nik Mohd Shah 2013; Iftekhar and Islam 2004; Sillanpää et al. 2017).

Trying to link these practices that affect mangrove stand age with the dynamics of organic matter-turnover, our understanding of potential drivers of leaf litter decay, such as plant species and age, is still limited. In terrestrial ecosystems, the availability of soil nutrients and turnover rates of organic matter decline as forests age (Gower et al. 1996; Ryan et al. 1997). Like other forest stands, mangroves follow a natural series of phases over time from an initial pioneering stage to rapid early growth and development, to later maturity, senescence and death (Jimenez et al. 1985). The aboveground net primary production (ANPP) reaches a peak early in forest stand development and then gradually declines as stands age (Berger et al. 2004; Gower et al. 1996; Alongi 2002; Alongi and Dixon 2000; Clough et al. 1999).

As a mangrove forest grows older, tree density declines naturally due to self-thinning, allowing for higher individual growth rates, and thus, providing higher aboveground biomass at the stand-scale with increasing age (Clough et al. 1999). In Northern Brazil, the annual leaf litter production steadily increases from early successional *Avicennia germinans* stands to late succession stages of *Rhizophora mangle*, while leaf litter quality steadily decreases (Quadros et al. 2019). Mature, old-grown mangrove stands rejuvenate naturally through the death of individual trees (Duke 2001) and lightning strikes (Zhang et al. 2008; Amir 2012), providing space and improved light conditions for dormant seedlings to replace the canopies of the dead trees. While such natural dynamics can be compared to man-made dynamics of forest management, the survival and recovery of mangrove forests are highly related to the scale of damage. In Honduras, mass mangrove tree mortality after severe storms caused rapid sediment elevation loss through the decay of dead root material and sediment compaction (Cahoon et al. 2003). Significant loss of organic C and N stocks was observed in a silvicultural mangrove stand of the MMFR, when compared to a reference site without any harvesting activities for more than 70 years (Adame et al. 2018).

A great proportion of the mangrove ANPP is shed as litter (Komiyama et al. 2008; Ong et al. 1995), which the leaf fraction being the largest contributor of total litter fall (Mfilinge et al. 2005; Nga et al. 2005). In general, plant litter decay and decomposition follow three main stages: (1) leaching of soluble components, (2) physical fragmentation and biological breakdown, and (3) microbial degradation of refractory components such as cellulose, hemicellulose, cutin and lignins (Valiela et al. 1985; Opsahl and Benner 1995; Zimmer 2019). Leaching accelerates litter degradation by promoting microbial activity and increasing the palatability of the litter to detritivores (Zimmer 2019). Fast decay and decomposition of detrital material with high N-content (or low C:N ratio) or low content of deterrent or recalcitrant compounds, such as phenolics, lignins or cellulose, have been associated with high rates of nutrient-turnover, rendering nutrients available for uptake by plants (and microbes) soon after litter fall. On the contrary, slowly decaying litter provides a longer-lasting pool of nutrients to be released into the soil or sediment environment more evenly over time. Upon burial into the belowground pool (for review, see Krishna and Mohan 2017), organic matter becomes more recalcitrant and degrades only slowly under saline and anoxic conditions (Kida et al. 2019). Further stabilization of organic matter is also induced by aggregation and strong chemical bonding to the mineral soil matrix (Cotrufo et al. 2013). Microbes influence the formation of organic matter through the transformation of plant residues by extracellular enzyme attacks and utilization of organic substrates via cell uptake. Insoluble structural carbohydrates and other complex biopolymers are subject to attack by microbial extracellular enzymes (Zimmer, 2019). Microbes convert the detrital organic matter into their own biomass, a process called immobilization, resulting in increasing amino acid and protein, as well as fatty acid and lipid contents of the detritus along with the decay processes (Mfilinge et al. 2003; Tremblay and Benner, 2006). Microbial immobilization generates compounds that are more stable or resistant against further degradation (Liang et al. 2017).

Much effort has been made to identify plant traits that drive decay and decomposition rates, an understanding of which is essential for accurate forecasts of the future carbon cycle. Cornwell et al. (2008) assigned leaf traits of different phylogenetic groups (e.g. N, P, lignins, leaf mass per area, water and acid soluble polysaccharides) to decomposition rates. In a study in India, the N content of yellow leaves was similar in the mangrove species *Rhizophora mucronata*, *R. apiculata*, *Sonneratia alba* and *Avicennia officinalis*, but the decomposition rate of *A. officinalis* was much higher than of the other three species (Wafar et al. 1997). In Malaysia, the litter of *S. alba* decomposed much faster than that of *Rhizophora* spp. and *Bruguiera parviflora* (Ashton et al. 1999). *Kandelia candel* litter decomposed faster than *Bruguiera gymnorhiza* litter in Japan, despite having a higher total phenolic content (Mfilinge et al. 2002); their senescent leaves differed markedly in initial N and P content, and C:N ratio. In Hong Kong, however, differences in decomposition rates between *Avicennia corniculatum* and *K. candel* were not explained by initial C:N ratios (Tam et al. 1990). The leaf litter of mangrove associates degrades at a higher rate than that of true mangroves (Chanda et al. 2016).

The content of phenolic compounds, specifically tannins, which are widely distributed among higher plants, is suggested to be a good predictor of decay, decomposition, nitrogen immobilization and mineralization (Maie et al. 2008). Rather than the phenolic content, however, the specific signature (fingerprint) of phenolic compounds can explain their biological activity (Zimmer et al. 2015). Tannins form recalcitrant complexes with proteins and thus impair decay processes through microbial extracellular enzymes (Kraus et al. 2003a). Members of the mangrove families Acanthaceae, Rhizophoraceae, and Sonneratiaceae are rich in tannins (Bandaranayake 1995), but tannin fate during decay in mangroves remains poorly understood (Constabel et al. 2014).

In particular, field studies on mangrove leaf litter decay and corresponding changes in leaf chemistry are still limited, even more so with respect to litter from herbaceous understory plants or to leaf litter derived from plants of different ontogenetic stages. Thus, through a field study on the microbial decay of leaf litter from woody versus herbaceous and immature versus mature plants, we test the hypotheses that (1) the initial chemical composition of the leaf litter of different species and maturity stages will determine decay processes; and (2) the microbial community engaged in decay will respond to the changes in leaf chemistry during decay by altering the activity of extracellular enzymes to break down recalcitrant litter compounds. We expect the findings of this study to contribute to our understanding of organic matter dynamics in mangrove stands of different compositions and ages, and thus, to develop into implications for mangrove management.

## Methods

### Study area and species

This study was conducted in the Matang Mangrove Forest Reserve (MMFR), located at the border of the Straits of Malacca on the northwest coast of Peninsular Malaysia (Fig. S1.1). The MMFR is a riverine mangrove forest that covers about 40,288 ha and is home to at least 27 mangrove species (Hamdan et al. 2014; Otero et al. 2019). However, more than 80% of the total area is occupied by the two commercially exploited species *Rhizophora apiculata* Blume and *Rhizophora mucronata* Lamk. (Ariffin and Nik Mohd Shah, 2013). The climate of the MMFR is year-round warm humid, with rainfall ranging from 2000 to 2800 mm year^−1^, average air temperature ranging from a maximum of 33° C during the day to a minimum of 22 °C at night, and the relative humidity averaging 99% in the early morning and 60% at midday (Muda and Mustafa 2003). The area is subjected to semidiurnal tides with the tidal amplitude range of 1.6–3 m (Goessens et al. 2014).

The decay experiment was performed near the Matang Mangrove Eco-Education Centre (4°50′31.4′′ N 100°38′08.2′′ E), at a site that had been reserved for educational purposes since 1998. Mangrove species other than *Rhizophora* spp. had been planted there to increase plant diversity. In 2020, the age of the mangrove forest in this area is estimated as 42 years. The selected mangrove species were the dominant species in the study area, which were divided into two categories: woody versus herbaceous plants, namely *Rhizophora apiculata* Blume and *Bruguiera parviflora* (Roxb.) Wight & Arn. ex Griff versus *Acrostichum aureum* L. and *Acanthus ilicifolius* L., respectively. Henceforth these species will be referred to by genus name.

### Experimental setup

Leaf litter decay was studied using litterbags with leaf litter from two ontogenetic stages of the above four species. Immature plants were identified by the absence of reproductive parts (e.g. flowers, propagules, fruits, fertile leaves with sporangia) and by the size and the volume of the plant, assuming these features are correlated with age (Sharpe and Mehltreter 2010). Mature plants were identified otherwise (e.g. diameter at breast height more than 20 cm for woody plants, higher plant volume for herbaceous plants and presence of reproductive parts). Yellowish senescent leaves for the experiment were either freshly fallen or easily hand-picked from the plants in the surrounding area. As senescent leaves of *A. aureum* are non-abscising, we hand-picked senescent leaflets (instead of entire fronds) from the plants.

Fresh senescent leaves were used in the litter decay experiment, as pre-drying accelerates the initial rate of mass loss through leaching (Ananda et al. 2008) and affects microbial activity. Twelve senescent leaves per bag were cut in half along the midrib and both halves were weighed. One half of each leaf was brought to the laboratory to obtain the dry:fresh weight ratio and the second half was utilized for the litterbag experiment. Litterbags were made of glass fibre mesh (200 mm × 200 mm, mesh size 1 mm^2^) and closed using polypropylene clips. The litterbag experiment started in November 2018 and lasted for 70 days, with an intermediate sampling of litterbags after 14 and 35 days. Another set of senescent leaves was sampled separately for determining initial leaf litter characteristics.

Seven replicate plots of 20 m diameter were distributed over an experimental site of 100 m × 100 m, with minimum distance among plots of 20 m (Fig. S1.2). The sediment of the MMFR is generally sulphur-rich (Priya et al. 2018) and fine-grained. Sediment pH, organic carbon content and total nitrogen content of this one-hectare site were 5.5 ± 0.5 (*n* = 7), 15 ± 5% and 0.6 ± 0.2% (*n* = 3), respectively. Litterbags were randomly placed at each plot on the sediment surface. On each sampling date, 56 litterbags of different species and ontogenetic stages were removed from the field and brought to the laboratory of the Biology Department of the Faculty of Science and Technology of the National University of Malaysia (FST UKM) for sample treatment and analysis.

The reduction of leaf litter mass over time was measured to obtain decay constants (*k*), leaf half-life (t_0.50_), and 95% and 99% lifespan (t_0.95_ and t_0.99_) (Eq. 1–4). Leaf material was cleaned carefully with a brush and tap water, then freeze-dried (Christ 1–2 LD Plus), and the final dry weight was recorded. Based on the dry:fresh weight ratio of the retained half leaves, their initial dry weight (X_o_) was estimated, and the difference to the remaining dry weight obtained from the litterbags after 14, 35 and 70 days (t) was used as mass loss upon decay (X_t_).

1 $${X}_{t}= {X}_{o }{e}^{-kt}$$

2 $${t}_{0.50}= \frac{ln (2)}{k}=\frac{0.6931}{k}$$

3 $${t}_{0.95}= \frac{3}{k}$$

4 $${t}_{0.99}= \frac{5}{k}$$

### Laboratory analyses

All laboratory analyses carried out in this study are summarized in Fig. S1.3. Fresh leaf litter subsamples were subjected to the measurement of microbial extracellular enzyme activities. Samples were weighed corresponding to 50–100 mg dry weight and cut into small pieces before they were homogenized with a tissue-tearer homogenizer (Dremel 395 MultiPro) in 10 mL of 0.10 M Na–K phosphate buffer on ice. Enzyme extraction was performed at (24 ± 2)  °C for 1 h in a rotary mixer (Elmi Intelli-Mixer RM-2L) at 90 rpm. The homogenates were centrifuged for 5 min, with 4000 g at 4 °C. The obtained supernatants were directly analysed or kept refrigerated (4 °C) until analysis within a few days. The enzymatic activity assays were analysed by UV–Vis spectrophotometer (Shimadzu UV-1800).

Phenol oxidase activity was determined through the spectrophotometric determination of 4-(*N*-proline)-*o*-benzoquinone resulting from enzymatic oxidation of catechol as the substrate in the presence of L-proline (adopted from Zimmer 2005b; Perucci et al. 2000). Upon mixing of 200 µL sample supernatant, 900 µL 0.05 M L-proline and 900 µL 0.05 M pyrocatechol, absorbance changes (*ΔA*) at 520 nm was recorded at 1-min intervals over 10 min. Relative catechol oxidation was determined as the slope of *ΔA* produced by linear regression analysis. Phenol oxidase activity was expressed as relative phenol oxidation capacity (RAU g^−1^ h^−1^).

Cellulase activity was quantified according to Zimmer (2005a), Skambracks and Zimmer (1998) and Linkins et al. (1990) with some modifications. A mix of 90 mg α-cellulose, 900 µL supernatant solution and 900 µL citrate–phosphate buffer (with 0.05% NaN_3_) was incubated in a rotary mixer for 18 to 24 h and then centrifuged for 15 min at 1000 g at room temperature. Blank solutions were prepared by mixing 700 µL supernatant with 300 µL citrate–phosphate buffer and measured at 340 nm to obtain absorbance at time zero (A_0_). For samples, 700 µL supernatant was mixed with 300 µL glucose (HK) assay kit solution (Sigma-Aldrich), incubated for 15 min at room temperature, and then the absorbance was measured (A_15_) at 340 nm. The final absorbance was calculated by subtracting A_0_ from A_15_ (ΔA).

Protease activity was determined through enzymatic decay of azocasein as a chromogenic protein substrate (after de Menezes et al. 2014; Charney and Tomarelli 1947). A mix of 500 µL supernatant solution and 500 µL 1% azocasein solution was incubated at room temperature for 1 h in a rotary mixer at 90 rpm. The proteolytic reaction was stopped by adding 500 µL 20% TCA to precipitate the remaining substrate. Then, the mixture was kept on ice for 10 min and centrifuged at 1000 g for 15 min at room temperature. For the blank solution, 20% TCA was added before the addition of sample supernatant. Both sample and blank solutions were alkalinized with an equal volume of 2 M NaOH solution prior to the photometric measurement at 440 nm. As the proteolysis of azocasein results in a mix of peptides and amino acids, the final result was expressed as relative protease activity (RAU g^−1^ h^−1^).

For leaf chemistry analyses –total carbon content, total nitrogen content, total phenolic content, protein-precipitation capacity and phenolic fingerprint– samples were shipped to the Leibniz Centre for Tropical Marine Research (ZMT), Bremen, Germany.

Approximately 1 to 2 g sub samples of fine-ground leaf-litter were weighed and encapsulated in 10 × 10 mm tin capsules for the measurement of carbon and nitrogen contents (Eurovector EA3000 Elemental Analyzer). Birch leaf standard (BLS) was used as standard. Another 50 mg of the samples were extracted with 2 mL of 70% ethanol at room temperature for 1 h with a rotary mixer at 90 rpm, and then the supernatants were separated from the precipitates by centrifugation at 10,000 g, 4 °C for 10 min.

The total phenolic content was determined following the Folin-Ciocalteu assay (Bärlocher and Graça 2005; Ainsworth and Gillespie 2007). Aliquots of 10 to 200 µL of the supernatants were diluted to 500 µL with double-distilled water. Then, 250 µL 1 N Folin-Ciocalteu reagent and 1.25 mL 700 mM Na_2_CO_3_ were added sequentially and the final solutions were vortexed. Blanks contained 500 µL double-distilled water but no sample. The mixture was kept in the dark for 1–2 h, before the absorbance was read at 750 nm using a microplate reader (TECAN Infinite M200 Pro). Tannic acid served as standard, and results are expressed as mg tannic acid equivalents (TAE) g^−1^ litter.

The protein-precipitation capacity of the leaf litter phenolics was quantified through a radial diffusion assay, using bovine serum albumin (0.1% in ascorbic acid-containing agarose) as protein (Graça and Bärlocher 2005; Hagerman 1987). Four consecutive 9 μL aliquots from each supernatant were added to a single 2 mm well in the agarose gel plate. Each plate contained three tannic acid standards of 0.09, 0.18 and 0.27 mg mL^−1^. The gel plates were incubated for 3 to 4 days at room temperature. Then, the average of two perpendicular diameters of protein precipitation rings from both standards and samples was determined using a stereomicroscope. The ring area was calculated after subtracting the diameter of the wells. Protein-precipitation capacity is expressed in TAE g^−1^ sample dry weight.

As the Folin-Ciocalteu and the radial diffusion assays provide different proxies for the phenolic signature of a sample (Zimmer et al. 2015), a phenolic fingerprint was additionally depicted through HPLC analysis of litter sample extracts. The supernatants of these extracts were dried in a multi-evaporator system (Heidolph Synthesis 1) for approximately 1 h, 1000 rpm, 60 °C. The pressure was set to 175 mbar for 30 min to evaporate ethanol and changed to 60 mbar to evaporate water. The dried extracts were dissolved with 2 mL acetonitrile mix reagent (23% acetonitrile:76% water:1% acetic acid v/v) in an ultrasonic bath for 10 min and filtered through 0.45 µm PTFE filters. The sample solutions were placed in 1.0 mL glass vials and kept frozen at -24^o^ C until analysis. Individual phenolic fingerprints of litter samples were obtained through reverse-phase high-performance liquid chromatography (Agilent 1260 Infinity), with a Luna 5 µm C18(2) non-polar column (100 Å, 250 × 4.6 mm) and detected at 280 nm with a Diode Array Detector. Flow-rate was kept at 1.25 mL/min, and temperature was set to 20 °C. From each sample, 20 µL was injected. The elution condition of the binary pump was: 0–11 min, 100% A (isocratic); 11–27.5 min, 75% A (linear gradient); 27.5–28.5 min, 0% A (linear gradient; 28.5–31 min, 0% A (isocratic); 31–32 min, 100% A (linear gradient); 32–35 min, 100% A (isocratic), with solvent A: 18% acetonitrile:0.5% acetic acid:81.5% water; solvent B: 100% acetonitrile. Gallic acid, tannic acid and sinapyl alcohol, a monomer of many lignins, served as standards for the identification and quantification of some of the resulting peaks.

Data and statistical analyses were conducted using the statistical package R version 3.4.4 (R Core Team 2018) and PAST software version 3.25 (Hammer et al. 2001). Normality of data was tested through Shapiro–Wilk’s tests and data were square root-transformed prior to the statistical analysis where necessary. The effects of ontogenetic and interspecific differences on leaf decay, leaf chemistry and extracellular enzyme activity over time were analysed through repeated measures analysis of variance (RM ANOVA) with fixed variations of species (4 levels), maturity stages (2 levels) and time intervals (4 levels). Bonferroni tests were used for post-hoc analyses (*α* = 0.05). Correlation coefficient matrices were created to determine the relationship of the leaf litter decay rate, represented as leaf half-life (*t*_0_._50_), with initial leaf chemistry and extracellular enzymatic activity variables.

Differences in phenolic fingerprints (HPLC analysis) were visualized through non-metric multidimensional scaling (NMDS) with Morisita-Horn similarity index (Magurran 2004). Non-parametric permutational multivariate ANOVA (PERMANOVA) with 9999 permutations was utilized to test for significance of interspecies and ontogenetic differences, based on the selected distance measure (Anderson 2001). Similarity Percentage (SIMPER) analysis was used for assessing which HPLC peaks (= phenolic compounds) were primarily responsible for an observed difference among groups of samples (Clarke 1993).

## Results

### Leaf litter decay

**Fig. 1:**
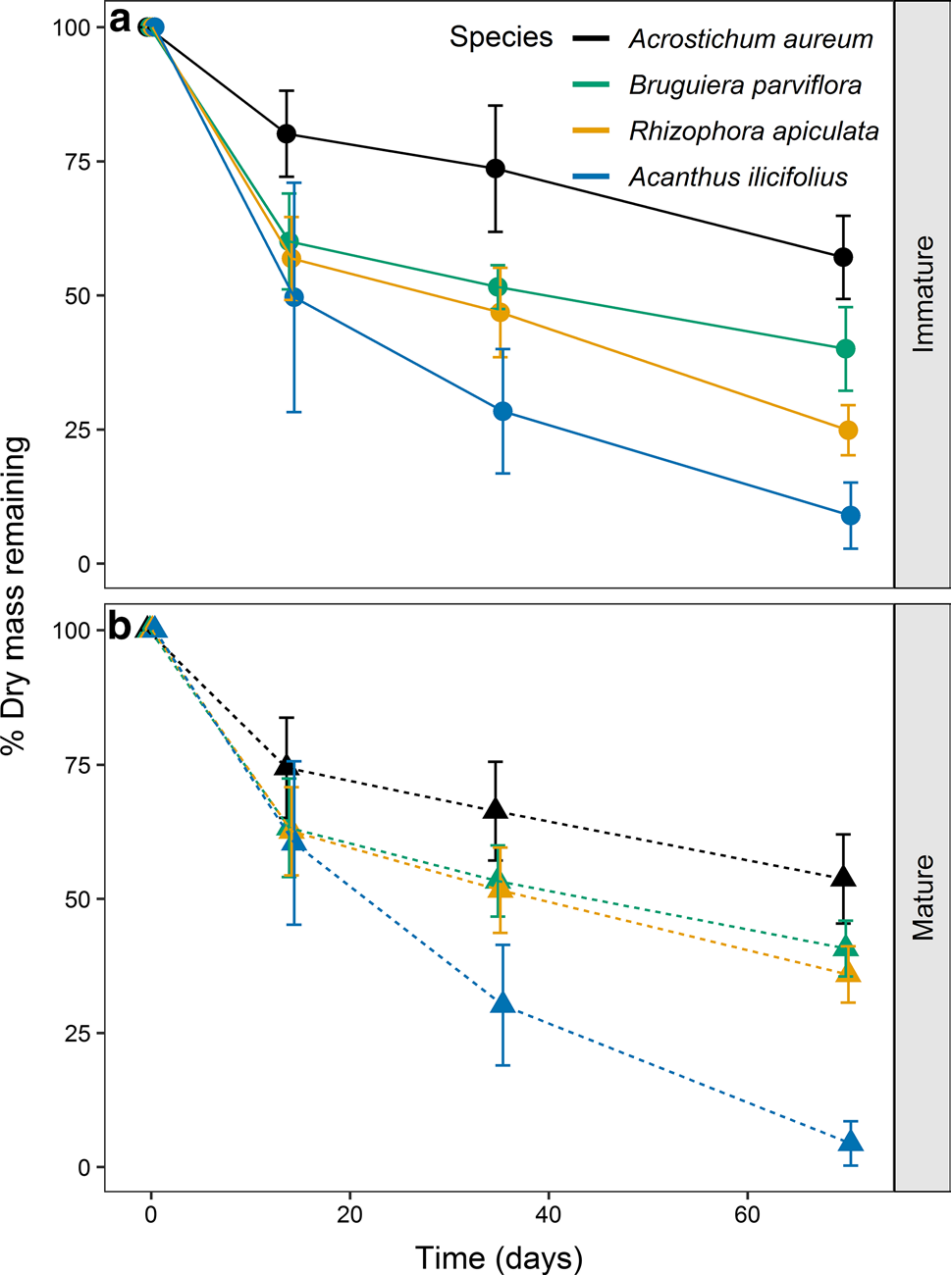
Remaining dry mass of leaf litter of different ontogeny and species overtime during the leaf litter decay experiments. Leaf litter decay ordered from the slowest to the fastest were *Acrostichum aureum* (black), *Bruguiera parviflora* (green), *Rhizophora apiculata* (yellow) and *Acanthus ilicifolius* (blue); **a** immature plants: circles; **b** mature plants: triangles. Error bars represent the 95% confidence interval of the mean (*n* = 224)

Leaf litter of all species exhibited an initial rapid mass loss during the first 14 days, followed by a slower mass loss during the following weeks (Fig. 1). Interspecific differences in the mass loss were more pronounced in the leaf litter of immature than mature plants. At the end of the decay experiment, the remaining mass proportion of *Acanthus* litter (4 ± 4% of initial mass) was significantly lower than of the other species, while the remaining mass proportion of *Acrostichum* litter was significantly higher than of the other species, especially for immature plants (57 ± 8%). The mass loss of *Rhizophora* and *Bruguiera* litter was more moderate than for the herbaceous species, with litter from immature *Rhizophora* (25 ± 5% remaining) decayed significantly faster than litter from immature *Bruguiera* (40 ± 8%) and mature *Rhizophora* (36 ± 5%). Decay patterns of all litter types fitted a single negative exponential model (*R*^2^ > 0.8) (Table 1). Litter of both immature and mature *Acrostichum* were projected to lose 99% of its mass after ~ 650 days, while *Acanthus* litter was projected to only need less than 5 months to decay almost completely (Table 1).

**Table 1:** Decay constant (k), half-life (*t*_0.50_), 95% and 99% lifespan (*t*_0.95_ and *t*_0.99_) of different mangrove species and maturity stages obtained from negative single exponential equations

Species name	Maturity stages	*R*^2^	Intercept	Decay constant –K (day^−1^)	*t*_0.50_ (days)	t_0.95_ (days)	t_0.99_ (days)
*Rhizophora apiculata*	Immature	0.950	87.649	0.0184	38	163	272
	Mature	0.918	87.014	0.0134	52	224	373
*Bruguiera parviflora*	Immature	0.839	83.835	0.0116	60	258	430
	Mature	0.876	85.951	0.0116	60	259	431
*Acrostichum aureum*	Immature	0.956	95.044	0.0074	93	403	672
	Mature	0.905	91.322	0.0081	86	370	617
*Acanthus ilicifolius*	Immature	0.991	90.371	0.0333	21	90	150
	Mature	0.984	112.81	0.0445	16	67	112

Species differed significantly from each other with respect to mass-loss rates (*p* < 0.0001; RM ANOVA: Table S2.1). We did not detect the overall effects of the ontogenetic stage, but upon within-species comparison, the litter of mature *Rhizophora* was projected to take about 40% longer to decay by 99% than that of immature *Rhizophora* (Table 1). According to a significant interaction of species x maturity (*p* = 0.028), the decay patterns of different ontogenetic stages changed over time.

### Leaf litter chemistry

**Fig. 2:**
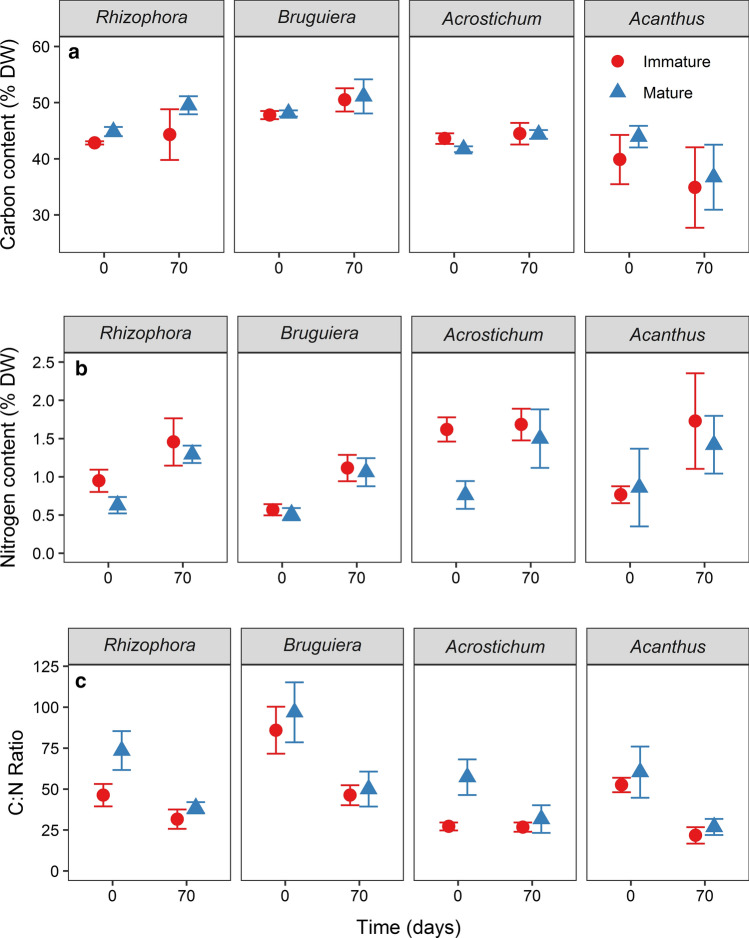
Mangrove leaf litter chemistry of **a** Total carbon contents, **b** total nitrogen contents, and **c** carbon-to-nitrogen (C:N) ratio of leaf litter from immature (circles) and mature (triangles) plants during the initial and final days of the decay experiment. Mangrove species *Rhizophora apiculata*, *Bruguiera parviflora*, *Acrostichum aureum,* and *Acanthus ilicifolius* are identified by their genus name. Error bars indicate 95% confidence interval of the mean (*n* = 112)

Litter carbon contents generally increased from the initial stage (Day 0) to the end (Day 70) of the decay experiments, except for *Acanthus* litter that exhibited decreased C contents after 70 days (Fig. 2a). The initial carbon contents of litter from immature *Rhizophora* plants were significantly lower than from mature *Rhizophora* plants. The litter with the highest initial carbon content was that of immature *Bruguiera* (48.1 ± 0.6%); litter of immature *Acanthus* had the lowest C content (40 ± 5%). We observed a significant interspecific differences of carbon contents (*p* = 0.011; RM ANOVA) (Table S2.2). Nitrogen contents of litter from immature plants were generally higher than those of litter from mature plants, being significantly different in *Rhizophora* and *Acrostichum* litter (Fig. 2b). The nitrogen contents of all species and ontogenetic stages increased significantly over the decay experiment (*p* < 0.0001) (Table S2.3).

Accordingly, initial C:N ratios of litter from immature *Rhizophora* and *Acrostichum* plants were significantly lower than from their mature plants (Fig. 2c). By the end of the decay experiment, the C:N ratio of all species and maturity stages had significantly decreased, except for litter from immature *Acrostichum.* The lowest initial C:N ratio was found in litter from immature *Acrostichum* (27 ± 3) and the highest in litter from mature *Bruguiera* (97 ± 20). In general, C:N ratios differed significantly among species and over time (*p* < 0.0001) (Table S2.4).

**Fig. 3:**
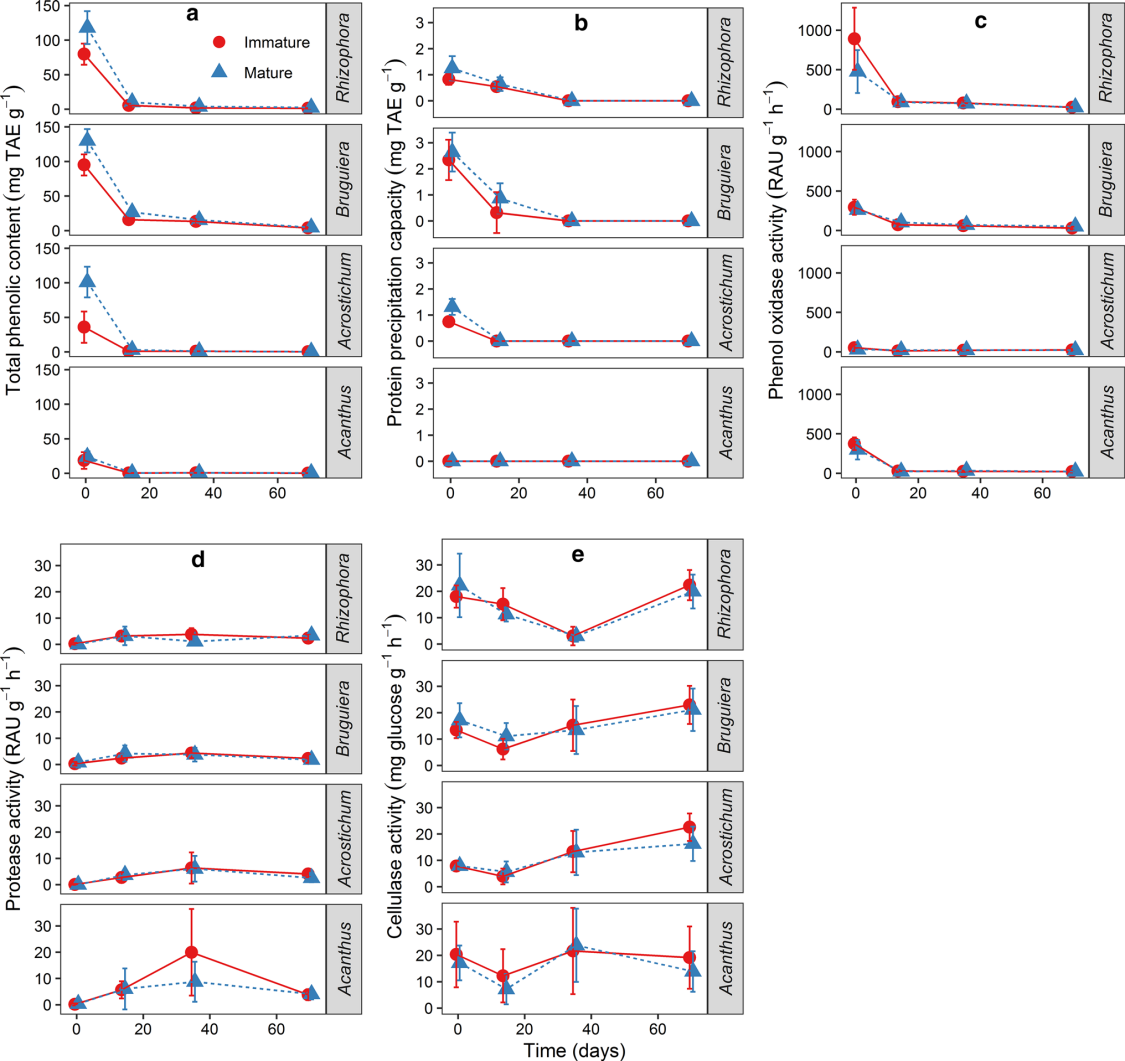
**a** Total phenolic contents, **b** protein precipitation capacity, **c** phenol oxidase, **d** protease, and **e** cellulase enzyme activity of mangrove leaf litter with different maturity stages and species at different time intervals (0, 14, 35, and 70 days); immature plants: circles; mature plants: triangles. Error bars indicate 95% confidence interval of the mean (*n* = 224)

*Bruguiera* litter was characterized by the highest content of phenolic compounds, followed by *Rhizophora*, *Acrostichum* and *Acanthus* (Fig. 3a). Litter of immature plants generally contained less phenolics than that of mature plants, but these differences were significant only for *Acrostichum* litter (Bonferroni post-hoc test *p* < 0.0001). The highest phenolic content was exhibited in senescent leaves (Day 0) and gradually decreased over time of decay. After 14 days of experiments, the phenolics were reduced sharply to around 20% or less of the initial contents. Significant interactions between species x time and maturity x time (Table S2.5) suggest species- and stage-specific patterns of changes in the phenolic contents.

Similar to the phenolic content, the protein-precipitation capacity of phenolic compounds was the highest in senescent leaves (Day 0) and steadily decreased over time (Fig. 3b). The litter of *Bruguiera* and *Rhizophora* had significantly higher protein-precipitation capacity than *Acanthus* litter, which exhibited no protein-precipitation capacity throughout the study. While the initial protein-precipitation capacity dropped drastically within the first days (Bonferroni post-hoc test *p* < 0.0001), there were no significant further changes during later phases of the experiment (*p* > 0.5) (Table S2.6).

HPLC analysis of phenolic signatures distinguished 38 peaks, representing unique phenolic compounds and their isomers (Figs. S3.1 and S3.2). Some of these peaks indicated specific biomarkers for either species or ontogenetic stages. Nine of the 38 detected peaks explained 57% of the cumulative differences among species and ontogenetic stages (SIMPER analysis, Table S3.1). These peaks, ordered from the highest to the lowest contribution, were Peak 22, 8, 10, 5, 4, 9, 11, 26, and 20. Peak 22 attributes to immature *Rhizophora*, while Peak 20 corresponds to mature *Rhizophora* (10.6 and 4.1% contribution, respectively). Peak 8 and 11 differentiate mature and immature *Bruguiera* litter (7.8 and 4.8% contribution). Peak 4 and 5 separate litter from mature and immature *Acrostichum* (6.4 and 6.9% contribution). Peak 9 was high in *Rhizophora*, *Bruguiera*, and *Acrostichum* litter but low in *Acanthus* that, in turn, was uniquely characterized by Peak 10 and 26 (7.2 and 4.4% contribution). Peak 26 was high in the herbaceous *Acrostichum* and *Acanthus* but low in the arboreal *Rhizophora* and *Bruguiera*.

Aiming at assigning these peaks to particular phenolic compounds, we used tannic acid and its monomer gallic acid, and the lignin monomer sinapyl alcohol, as well as d-glucose as one of the most common components of sugar esters in tannic acid and other compounds, as standards. Peak 6, 11, 22 and 28 are the major peaks (Table S3.1), of which Peak 6 represents gallic acid, of the complex mix of tannic acid. Sinapyl alcohol is reflected by Peak 20, while Peak 8 represents d-glucose.

According to the above standard compounds, the major phenolic difference between *Acanthus* and the other species was the lack of gallic acid and its galloyl esters. The diversity of phenolic compounds in *Rhizophora* leaves was low and characterized by a lack of compounds with higher retention times, possibly reflecting compounds with low hydrophilicity. The relative contribution of different phenolic compounds was significantly different among both species and ontogenetic stages (PERMANOVA: *p* < 0.001) (Table 2). The significant interaction between species x maturity indicates species-specific effects of the ontogenetic stage on the phenolic signature.

**Table 2:** PERMANOVA results of the effects of interspecific and ontogenetic (maturity stages) differences on the phenolic signatures of mangrove leaf litter

Source of difference	Sum of squares	*df*	Mean square	*F*	*P* value
Species	1.7755	3	0.59183	23.277	< 0.001
Maturity	0.15795	1	0.15795	6.2122	< 0.001
Interaction	0.38644	3	0.12881	5.0663	< 0.001
Residual	1.2459	49	0.025426		
Total	3.5658	56			

### Extracellular enzyme activity

Phenolic contents of litter were highest, and phenol oxidases exhibited the highest activities, during the initial stage of decay, and both constantly decreased over time (Fig. 3c). Phenol oxidase activity changed significantly over time within species (RM ANOVA *p* < 0.0001: Table S2.7). The activity of phenol oxidases was highest in *Rhizophora* litter, and higher in litter from mature than immature plants (Bonferroni post-hoc test *p* = 0.0003). Significant ontogenetic differences were also found in *Bruguiera* litter (*p* < 0.0001).

Hardly any protease activity was detectable at Day 0 (Fig. 3d), being lower in *Rhizophora* and *Bruguiera* than in *Acrostichum* and *Acanthus* litter (*p* < 0.0001). The ontogenetic stage had a significant influence (*p* = 0.005), too, and protease activity changed significantly over time, generally peaking at Day 35 (Table S2.8).

Cellulase activity decreased over the first 14–35 days and then generally increased again until the end of the experiment (Fig. 3e). By contrast, the decreasing cellulase activity in *Acanthus* litter may be attributed to the high mass loss of litter from this species. Differences among ontogenetic stages were negligible (Table S2.9).

## Discussion

Mass loss, as well as chemistry and enzyme activities, of litter from different species and ontogenetic stages of mangrove plants changed over time upon microbial decay following the typical three phases of decay, namely leaching, fragmentation and degradation phases (Zimmer 2019). Overall, litter half-life was positively correlated with total phenolic content and protein-precipitation capacity, and negatively correlated with phenol oxidase and cellulase activity. Many studies relate high decay rates to low C:N ratio (high N content) and low contents of lignins and cellulose (e.g. Hernes et al. 2001; Bosire et al. 2005), but these variables did not exhibit any significant effect on decay rates in the present study. Thus, leaf phenolics slowed down litter decay, while the microbial activity of phenol oxidases and cellulases accelerated decay processes, and these parameters may prove better predictors of decay rates of mangrove litter than those mentioned before. Hence, our study supports the hypothesis of Cotrufo et al. (2013) that plant litter with high contents of labile low-molecular weight compounds is utilized more efficiently by microbes than recalcitrant plant litter. During decay processes, microbial immobilization of N into biomass, together with more excessive leaching of C- than N-containing compounds, results in (relative) increase in the litter N content over time (Schimel and Bennett 2004). In the present study, the N contents of all species and ontogenetic stages increased over the 70 days of the experiment. Substrates with a low C:N ratio (i.e., relatively higher N content) generally support higher microbial abundances and higher microbial decay rates (Bouillon et al. 2004; Manzoni et al. 2008). While it was previously assumed that the accumulation of recalcitrant litter contributes more to organic matter formation in sediment than labile fast-decaying litter (e.g. Freschet et al. 2012), our results suggest that labile litter is the dominant source of microbial products that, in turn, are the largest contributor to the build-up of sediment organic matter (Gleixner 2013; Cotrufo et al. 2013).

**Fig. 4:**
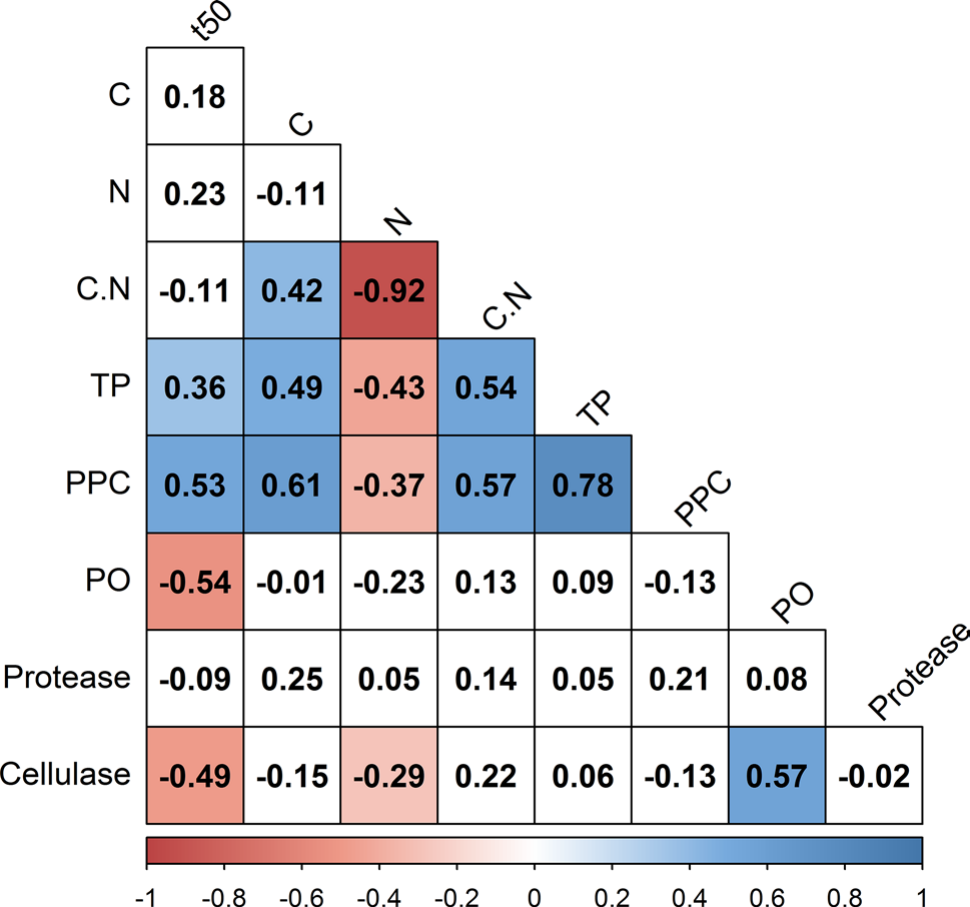
Heat map of correlation coefficients between litter half-life (*t*_0.50_) and initial litter characteristics. Variables that correlate significantly at *α* = 0.05 are marked blue for positive and red for negative correlation (*n* = 56). Fields with non-significant correlation coefficients are left blank. *C*  carbon, *N* nitrogen, *C.N* C-to-N ratio, *TP* total phenolics, *PPC* protein precipitation capacity, *PO*  phenol oxidase

Our quantitative proxies of the phenolic signature of the litter were correlated negatively with N and positively with C (Fig. 4). This observation is consistent with the two strategies of resource allocation of plant defence against herbivory and pathogens, either by producing highly recalcitrant C-rich compounds, such as phenolics and terpenoids or by producing N-rich compounds, such as alkaloids and glucosinolates, that are often not recalcitrant but deterrent or toxic (Wittstock and Gershenzon 2002; Schultz et al. 2013). From our observation, it is possible that the allocation of defence chemicals as either C-rich compounds or N-rich compounds changes during the ontogenetic development in mangrove species, and thus, differs between immature and mature plants. Therefore, understanding differences and changes in allocation strategies is relevant to predict organic matter turnover and dynamics which, in turn, are key elements of storing climate-active gases in sediment organic matter ("blue carbon").

Phenol oxidase activity on litter from all plant species and ontogenetic stages showed a significant decrease over the time of decay, along with the leaching-driven decrease in phenolic content. Microbial enzymes, such as phenol oxidase and cellulases, are the major drivers of decay (Allison et al. 2007; Sinsabaugh et al. 2008). The activities of phenol oxidases and cellulases were positively correlated with each other, suggesting synergistic action during microbial decay, as some phenolics are bound to cellulose and other plant polymers (Zhou et al. 2012). On the other hand, both enzyme activities were negatively correlated with the N content, corroborating the observation of Saiya-Cork et al. (2002) in terrestrial forests that the decomposition of recalcitrant soil organic matter is inhibited by N in the environment, possibly through a reduced need for degrading macromolecules when N is available. This correlation might help explain that the influence of N content or C:N ratio on decay rates was much weaker (and non-significant) than that of phenolic contents and the activities of cellulases and phenol oxidases.

The initial leaching of soluble compounds resulted in a rapid reduction of litter mass, content of phenolics, protein-precipitation capacity and phenol oxidase activity during the first days of decay (Li and Ye 2014; Zimmer 2019). The release of soluble compounds into the sediment (porewater) results in an accumulation of insoluble, i.e., recalcitrant high-molecular-weight compounds, such as condensed tannins, cellulose and lignins, in the litter. When the lignin content of litter is below 10%, other factors limit decay, but at lignin contents of above 28%, decay rates will be uniformly low because of the recalcitrant nature of lignins (Prescott 2005). Thus, while N-immobilization accelerates decay, the accumulation of recalcitrant C-rich compounds slows down decay and results in a shift of the microbial community from generalists towards specialists (Das et al. 2007).

Microbial colonization commences long before leaf abscission, essentially by microbial endophytes or pathogens of the living leaf (Hardoim et al. 2015). Especially pathogenic fungi colonizing photosynthetically active leaves might, thus, be responsible for high initial phenol oxidase and cellulase activities (Arfi et al. 2012) and contribute to initial microbial decay processes. The increase in protease activity of the first 2–3 weeks of decay suggests an increase of protein substrates, such as in microbial biomass upon N-immobilization, as many fungi produce particular enzymes only upon demand, i.e., when required for particular substrates (Schimel and Weintraub 2003).

### Interspecific differences in decay rates

**Fig. 5:**
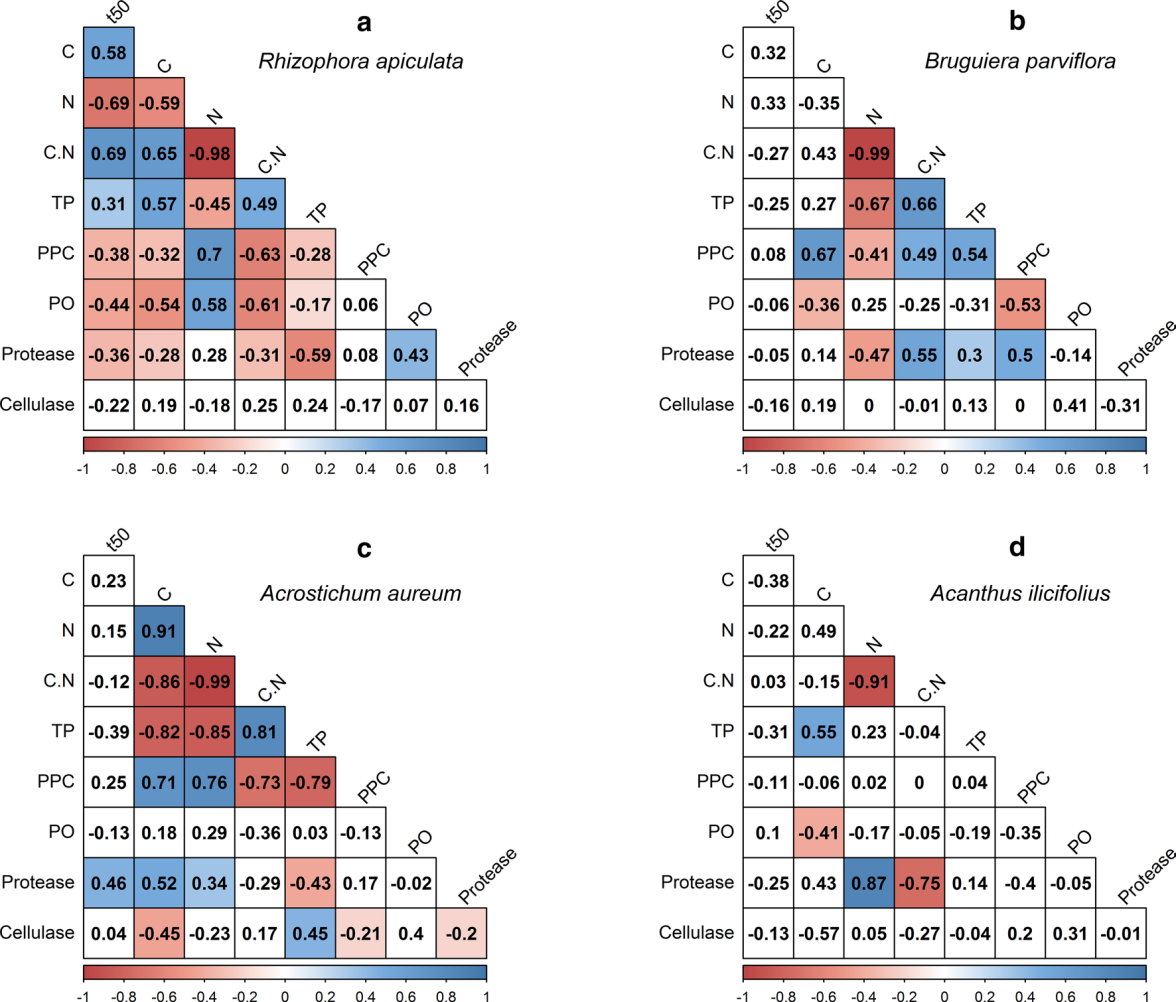
Heat maps of correlation coefficients between leaf half-life (*t*_0.50_) of **a** *Rhizophora apiculata*, **b** *Bruguiera parviflora*, **c** *Acrostichum aureum*, **d** *Acanthus ilicifolius*, and initial litter characteristics at *α* = 0.05 (*n* for each species = 14). *C*  carbon, *N*  nitrogen, *C.N* C-to-N ratio, *TP* total phenolics, *PPC* protein precipitation capacity, PO = phenol oxidase

The decay pattern of leaf litter of all species followed a single exponential function, only differing in the mass remaining over time. The kinetics of decay (decay constants, leaf half-life and 95% lifespan) observed in this study are comparable to findings by Ashton et al. (1999), Chanda et al. (2016), and Kamal et al. (2020) (Table 3). Leaf litter was observed to disintegrate physically and fragment within 14 days for *Acanthus*, within 35 days for *Rhizophora* and *Bruguiera*, and within 70 days for *Acrostichum*. Similar to a previous study by Ashton et al. (1999), litter of *Rhizophora* decayed faster than that of *Bruguiera*. Along the same line, significant interspecific differences were observed in litter chemistry and enzyme activities, as well as in their respective patterns of change over time. Accordingly, pair-wise correlations of these litter characteristics with decay kinetics (litter half-life) suggested those parameters that drive decay rates in general (Fig. 4), as well as interspecific differences in decay rates (Fig. 5). It is mostly for the decay of *Rhizophora* litter that the parameters we measured correlate with litter half-life.

**Table 3:** Comparison of decay constants (k), goodness of fit (*R*^2^), leaf half-life and 95% lifespan of this study and other studies performed in Asian mangroves. IM and M differentiate immature and mature plants

Mangrove species	Location	Decay constant, (day^−1^)	*R*^2^	*t*_0.50_ (days)	*t*_0.95_ (days)	References
*Rhizophora apiculata*	Matang, Malaysia	0.0091 – 0.0163	0.635 – 0.884	43 – 76	–	Ashton et al. (1999)
	Sundarbans, Bangladesh	0.13 – 0.14	0.587 – 0.675	35 – 37	–	Chanda et al. (2016)
	Sibuti, Malaysia	0.013 – 0.0103	0.728 – 0.981	55 – 68	238 – 291	(Kamal et al. 2020)
	Matang, Malaysia	0.0184 (IM) 0.0134 (M)	0.950 (IM) 0.918 (M)	38 (IM) 52 (M)	163 (IM) 224 (M)	This study
*Bruguiera parviflora*	Matang, Malaysia	0.0057 – 0.0099	0.618 – 0.747	70 – 122	–	Ashton et al. (1999)
	Matang, Malaysia	0.0116 (IM & M)	0.839 (IM) 0.876 (M)	60 (IM & M)	258 (IM) – 259 (M)	This study
*Bruguiera gymnorhiza*	Okinawa, Japan	0.022	0.993	32	–	Mfilinge et al. (2002)
*Nypa fructicans*	Sundarbans, Bangladesh	0.08 – 0.10	0.489 – 0.730	23 – 61	–	Chanda et al. (2016)
*Acrostichum aureum*	Matang, Malaysia	0.0081 (IM) 0.0074 (M)	0.956 (IM) 0.905 (M)	93 (IM) 86 (M)	403 (IM) 370 (M)	This study
*Acanthus volubilis*	Sundarbans, Bangladesh	0.21 – 0.29	0.606 – 0.701	17 – 23	–	Chanda et al. (2016)
*Acanthus ilicifolius*	Matang, Malaysia	0.0333 (IM) 0.0445 (M)	0.991 (IM) 0.984 (M)	21 (IM) 16 (M)	90 (IM) 67 (M)	This study

Besides purely quantitative differences among species and between ontogenetic stages (see below), qualitative differences in the phenolic signature appear to remarkably contribute to the explanation of decay patterns. Some dominant phenolic compounds of *Rhizophora* litter (Peaks 20–22; Fig. S3.1–2) were not, or only in small amounts, detected in other species. The dominant Peak 22 in the litter of immature *Rhizophora* and its lack in the litter from mature *Rhizophora* may be attributed to a specific phenolic compound that is produced during the early growth stages of this species and drives differences in decay rates between immature and mature *Rhizophora*. It remains unclear, though, how the dominance of these two peaks may drive the faster decay of *Rhizophora* litter compared to that of *Bruguiera* and *Acrostichum*. Peaks 11 and 12 were specific to *Bruguiera* and may thus explain partly the differences between the latter and *Rhizophora*. The lack or low contents of Peaks 6 and 20–22 in *Acanthus* might partly explain the significantly higher decay rates of this herbaceous species. On the other hand, a higher diversity of phenolic compounds in *Bruguiera* and *Acrostichum* litter may contribute to the deceleration of microbial activity during the early phase of decay.

Overall, the very low content of phenolics in *Acanthus* did not exhibit any measurable protein-precipitation capacity, possibly explaining the lack of effect of phenolics in *Acanthus* on microbial decay rates. Additional species-specific parameters probably also drive differences in decay rates, but among those we quantified herein, only the ones related to phenolic compounds and enzyme activity proved significant, whereas traditional predictors of decay rates, such as N content or C:N ratio, had only little explanatory power. Even though the C:N ratio of litter from *Bruguiera* was higher than that of the litter from *Acrostichum,* the former decayed faster than the latter*.* Some N compounds, such as alkaloids, are deterrent or even toxic for microorganisms. *Acrostichum aureum* was tested positive for alkaloids (Basyuni et al. 2019), and it contains abundant sterols, flavonoids, fatty acids and long-chain hydrocarbons (e.g. sesquiterpenes) (Uddin et al. 2011) that might be responsible for the slow decay of this species in our study.

### Ontogenetic differences in decay rates

**Fig. 6:**
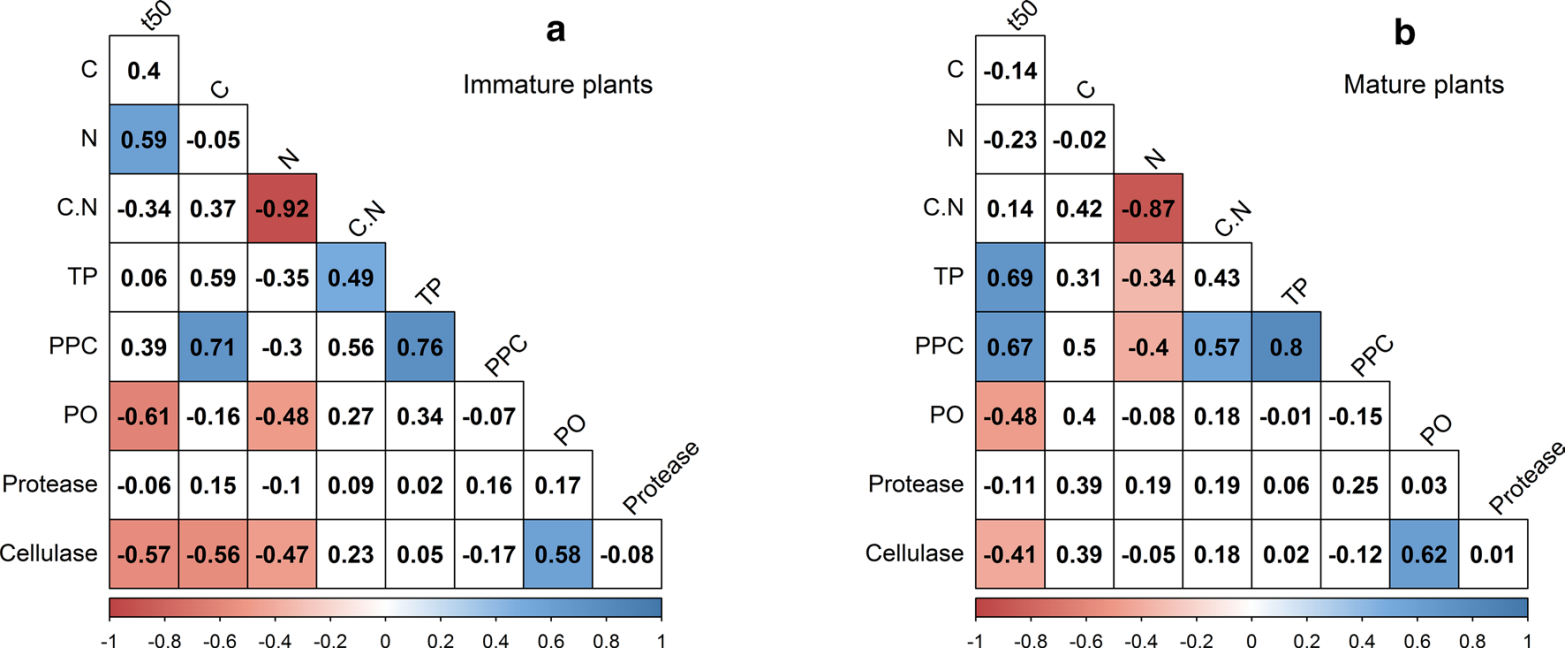
Heat maps of correlation coefficients between leaf half-life (*t*_50_) of **a** immature plants and **b** mature plants, and initial litter characteristics at *α* = 0.05 (*n* for each maturity stages = 28). *C* carbon, *N* nitrogen, *C:N*  C-to-N ratio, *TP* total phenolics, *PPC*  protein precipitation capacity, *PO* phenol oxidase

From the set of parameters we measured herein, total phenolic content, protein precipitation capacity, and phenol oxidase and cellulase activity significantly explained the variance in litter half-life for litter from mature plants (Fig. 6), which followed the correlation matrix of the entire data set (Fig. 4). For the litter from immature plants, we observed significant correlations between litter half-life and N content, phenol oxidase and cellulase activity. In general, the half-life of immature leaves was significantly decreased when initial N content and initial activities of phenol oxidases and cellulases were high.

**Fig. 7:**
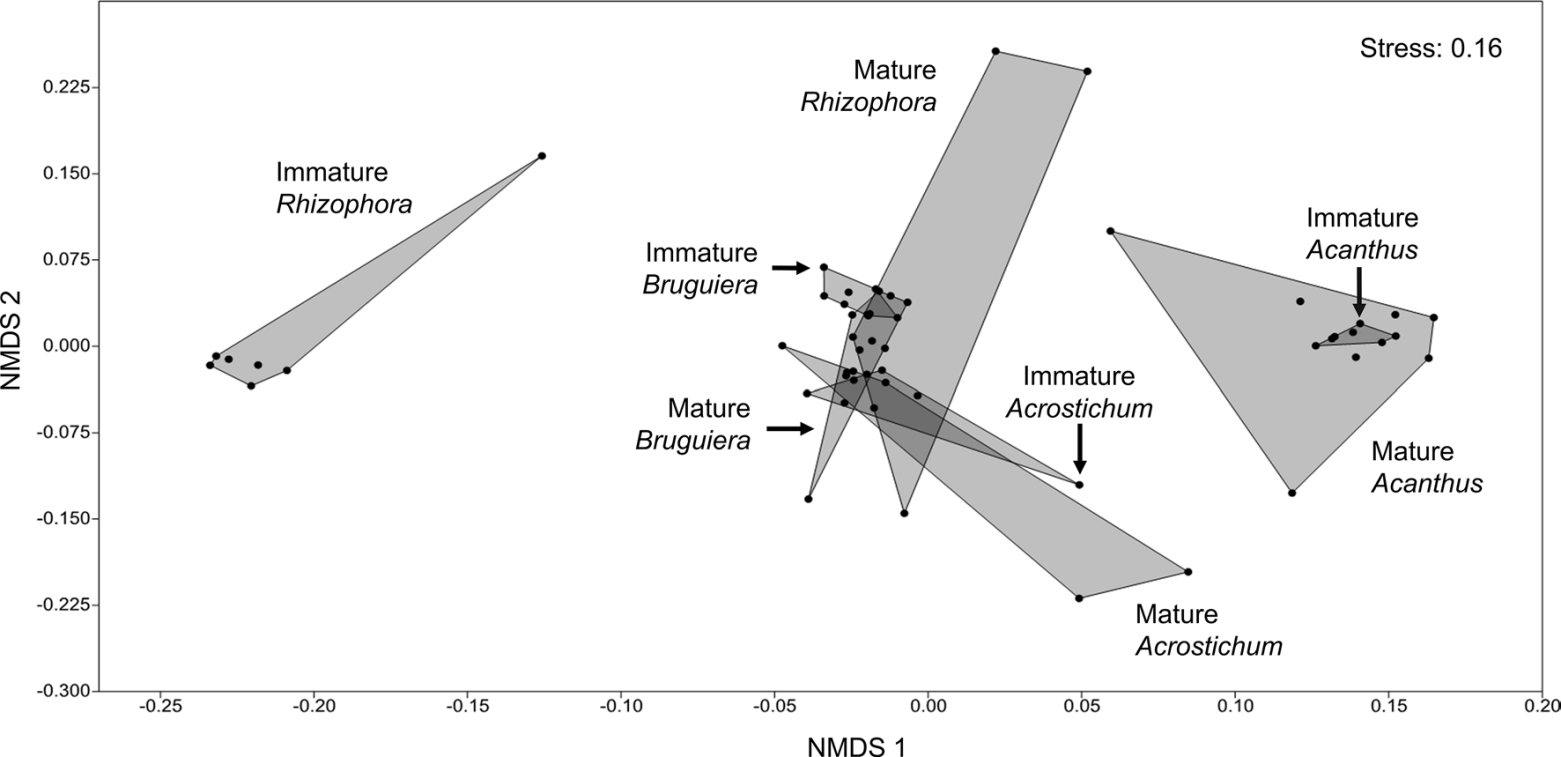
Non-metric multidimensional scaling (NMDS) plot of phenolic signatures of individual plants with different species and maturity stages grouped into different convex hulls. IM and M differentiate immature and mature plants

Our observations indicate that ontogenetic differences in the content and protein-precipitation capacity of phenolic compounds affect the variance in decay rates, as these quantitative measures of the phenolic signature were significantly different in mature plants but not in immature plants. Structural characteristics of foliar phenolics, especially tannins, can vary significantly among closely related species or even varieties and also may vary by physiological stage, season, and environmental conditions (Kraus et al. 2003b; Lin et al. 2006; Zimmer et al. 2015). The qualitative analysis of the phenolic fingerprints of different litter types yielded a clear pattern in that litter from immature *Rhizophora* had a clearly distinct phenolic fingerprint from the litter of all mature plants (Fig. 7). Litter from mature *Rhizophora*, by contrast, exhibited high similarity to the litter from both immature and mature *Bruguiera* and *Acrostichum*. Corresponding with the generally low phenolic content and the lack of protein-precipitation capacity of *Acanthus* litter, this species was clearly separated from the other species regarding the phenolic fingerprint. Compared to other species, the dissimilarity of phenolic signatures of litter from immature *Rhizophora* was clearly shown in Peak 22 (49% of total peak area) that was lacking from a litter from mature *Rhizophora* and had very low contents (< 2% of total peak area) in other species (Table S3.1). On the other hand, the phenolic fingerprint of litter from mature *Rhizophora* was different from that of litter from immature plants and other species in Peak 20 (17%).

Despite the clear differences in litter chemistry between ontogenetic stages of the same species, we detected only weak effects of the ontogenetic stage on decay rates. However, the within-species comparison demonstrated that litter from mature *Rhizophora* had a by about 40% increased lifetime (99%) compared to litter from immature plants of the same species. Faster decay of litter from immature *Rhizophora* can be interpreted as (1) faster release of CO_2_ upon microbial decay, but at the same time (2) increased release of organic matter into the sediment and porewater where it will be microbially immobilized and transformed into recalcitrant humic substances and stored under anoxic conditions for decades and centuries (Kida et al. 2019). While silivicultural mangrove stands lose parts of their C- and N-stocks upon clear-felling (Adame et al. 2018), we hold that the actual sequestration rate of C into the sediment might be higher upon reforestation of clear-cut areas underneath immature fast-growing *Rhizophora* trees than under mature trees with low relative growth rates. Further studies are needed to confirm this hypothesis.

### Implications for mangrove forest management

According to the findings of the present study, changes in species composition of mangroves over time, such as observed over the successional stage after disturbance, will result in changes in litter chemistry, and thus, decay rates (c.f. Quadros et al. 2019) which, in turn, will translate into changes in storage rates and eventually stores of organic matter in the sediment. The common assumption that high initial N content and low C:N ratio influence the fast decay of mangrove leaf litter, however, is not corroborated by the findings of this study. Significant differences in the phenolic signature of leaves from immature vs mature *Rhizophora*, on the other hand, seem to drive the faster decay of litter from immature plants. It remains an open question as to whether the differences in decay rates of litter from mature vs immature *Rhizophora* increase CO_2_ release into the atmosphere or foster the storage of organic matter in the sediment. The answer to this question will help us understand what spatially limited clearcutting of mangrove patches means for organic matter turnover dynamics and organic matter stocks in the sediment. Frequent rejuvenation of mangrove stands through controlled and spatially limited clearcutting and long-term use of logs for construction, rather than using them as firewood or processing them into charcoal, might prove an efficient tool for increasing the blue carbon-storage efficiency of mangrove stands.

## Supplementary Information

Below is the link to the electronic supplementary material.

Supplementary file1 (DOCX 3613 KB)
