# Correction to: Plant species- and stage-specific differences in microbial decay of mangrove leaf litter: the older the better?

**DOI:** 10.1007/s00442-021-04887-x

**Published:** 2021-03-15

**Authors:** Novia Arinda Pradisty, A. Aldrie Amir, Martin Zimmer

**Affiliations:** 1 Faculty 2 Biology/Chemistry, Universität Bremen, Bremen, Germany; 2 Institute for Marine Research and Observation, Ministry of Marine Affairs and Fisheries, Negara, Bali Indonesia; 3 Institute for Environment and Development (LESTARI), Universiti Kebangsaan Malaysia, Bangi, Selangor Malaysia; 4 IUCN SSC Mangrove Specialist Group, London, England; 5 Leibniz Centre for Tropical Marine Research, ZMT-GmbH, Bremen, Germany

## Correction to: Oecologia 10.1007/s00442-021-04865-3

Authors would like to correct the errors in their publication.

The original article has been corrected.

Incorrect Supplementary Information (ESM2) deleted now.

The line “A color version of this figure is available online” to be deleted from all figure captions since the color figures in print version is not charged now.

Missing references for the text citations were updated here

Alongi, 2002

Alongi DM (2002) Present State and Future of the World’s Mangrove Forests. Environ Conserv 29:331–349. 10.1017/S0376892902000231

Alongi and Dixon, 2000

Alongi DM, Dixon P (2000) Mangrove primary production and above-and below-ground biomass in Sawi Bay, southern Thailand. Phuket Mar Biol Cent Spec Publ 22:31-38.

Arfi et al. 2012

Arfi Y, Buée M, Marchand C, et al (2012) Multiple markers pyrosequencing reveals highly diverse and host-specific fungal communities on the mangrove trees *Avicennia marina* and *Rhizophora stylosa*. FEMS Microbiol Ecol 79:433–444. 10.1111/j.1574-6941.2011.01236.x

Krishna and Mohan, 2017

Krishna MP, Mohan M (2017) Litter decomposition in forest ecosystems: a review. Energy, Ecol Environ 2:236–249. https://doi.org/10.1007/s40974-017-0064-9

Murdiyarso et al. 2015

Murdiyarso D, Purbopuspito J, Kauffman JB, et al (2015) The potential of Indonesian mangrove forests for global climate change mitigation. Nat Clim Chang 5:1089–1092. 10.1038/nclimate2734

Quadros et al. 2019

Quadros AF, Nordhaus I, Reuter H, Zimmer M (2019) Modelling of mangrove annual leaf litterfall with emphasis on the role of vegetation structure. Estuar Coast Shelf Sci 218:292–299. 10.1016/j.ecss.2018.12.012

