# Supplementary material for: Plant species- and stage-specific differences in microbial decay of mangrove leaf litter: the older the better?
Source: Oecologia. 2021 Feb 9;195(4):843–58. doi: 10.1007/s00442-021-04865-3 (PMC8052233; doi:10.1007/s00442-021-04865-3)
Supplement: Supplementary file 1 — Supplementary file1 (DOCX 3613 KB) [file 442_2021_4865_MOESM1_ESM.docx]

**Supplementary Material 1.** Field experimental setup and laboratory analyses of this study

| 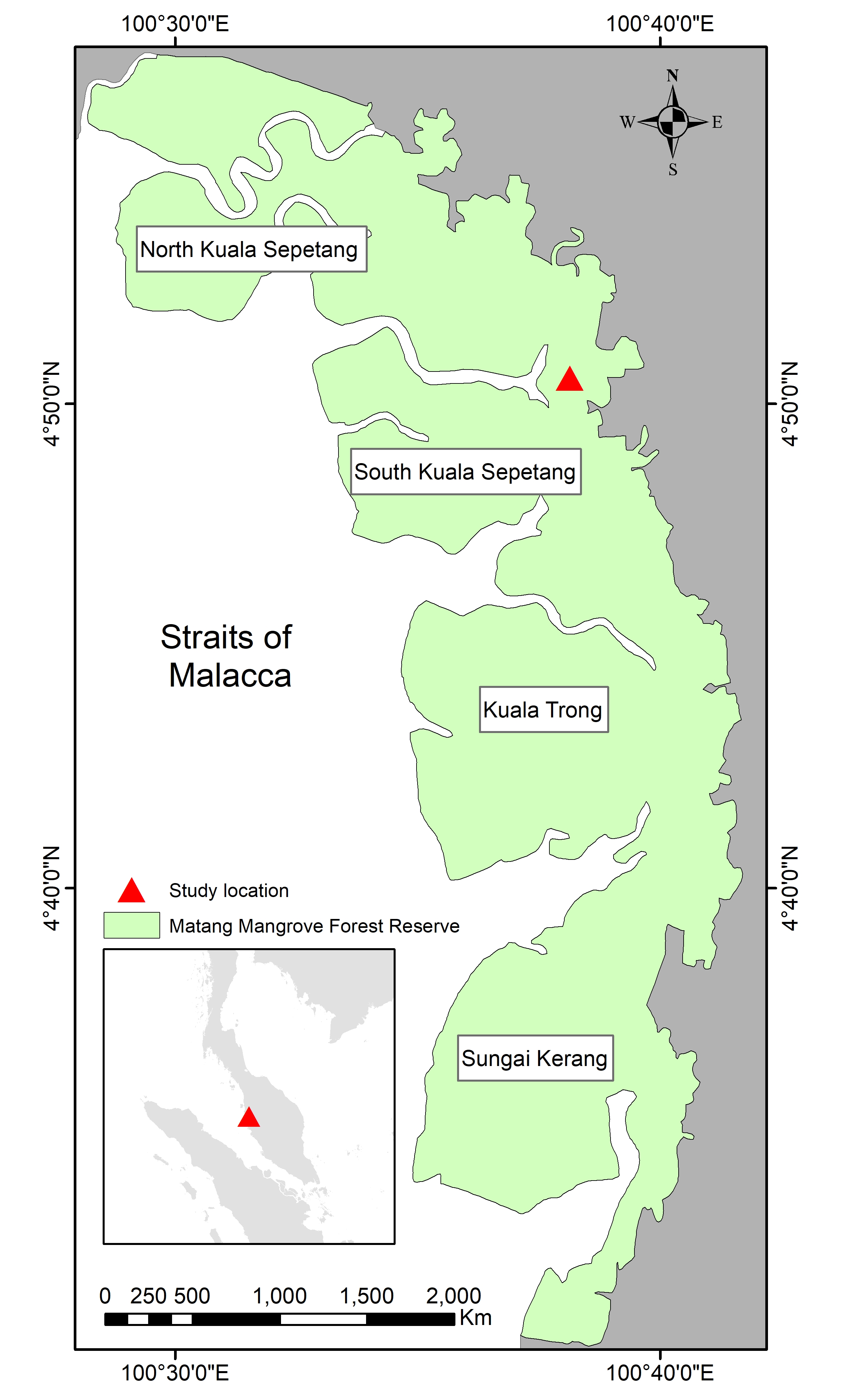 |  |
| --- | --- |
| **Figure S1.1** Map of Matang Mangrove Forest Reserve in Peninsular Malaysia, divided by 4 sub-areas (Kuala Sepetang – North and South, Kuala Trong, and Sungai Kerang) and study location (triangle). |  |
| 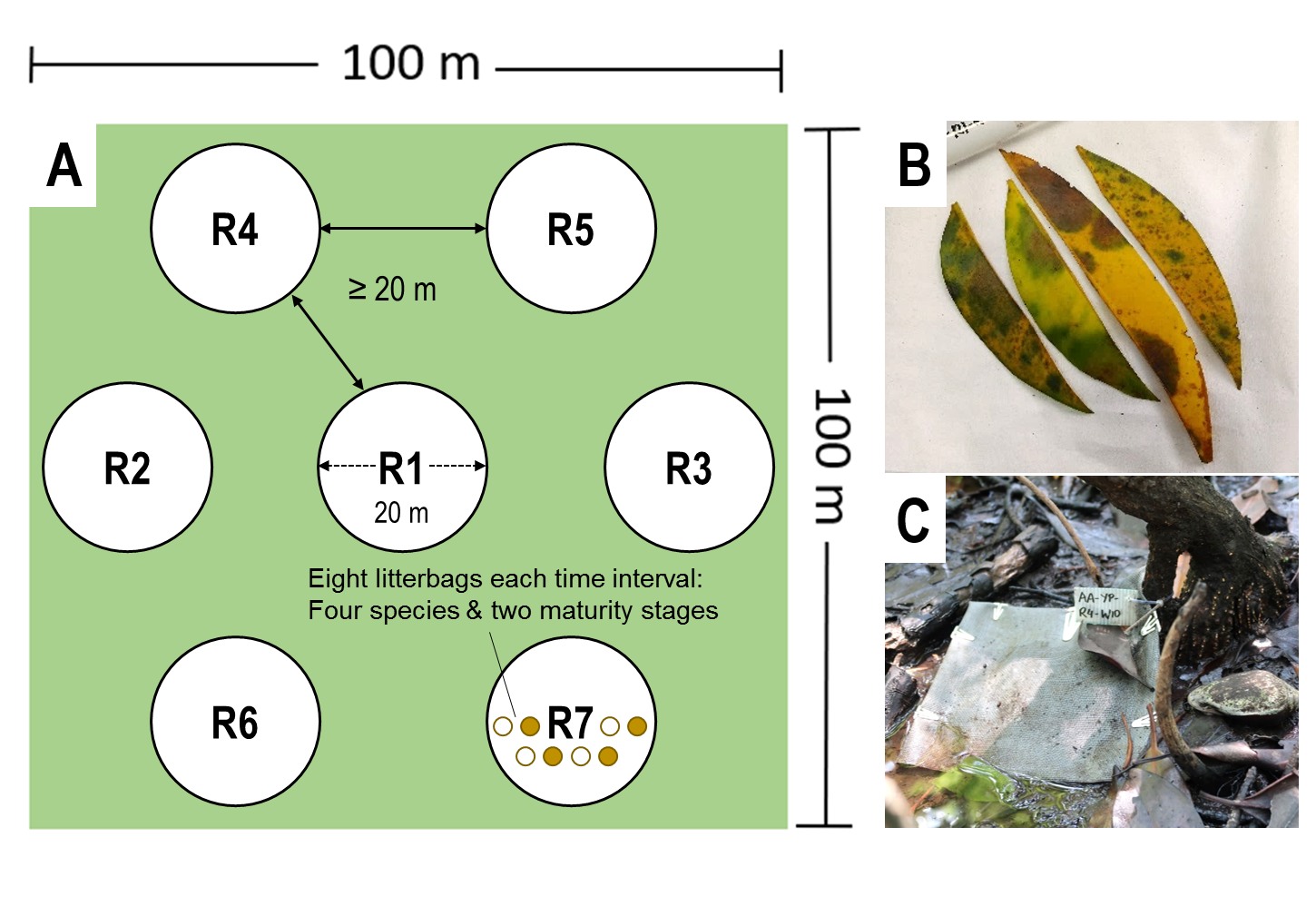 | |
| **Figure S1.2 (A)** Experimental setup of this study. Seven 20 m-diameter replicate plots were located within 1-hectare experimental site, with distance between plots of ≥ 20 m. Each litter bag consists of different mangrove species (4 levels), maturity stages (2 levels) and time intervals (4 levels). Different phases of leaf litter decay phase were investigated at 0, 14, 35 and 70 days, to give 224 total sample size. Day 0 samples were collected separately. About 24 litterbags were assigned to random positions inside the plots and eight bags were taken at each sampling time**. (B)** Example of leaf litter sample preparation**,** where in this picture senescent *Bruguiera parviflora* leaves were cut in half along the midrib to obtain the exact dry:fresh ratio for each of the samples **(C)** During the leaf decay experiment, the litterbag was tied to *Rhizophora* spp. or *Bruguiera* spp. roots by nylon threads or cable ties. | |

| 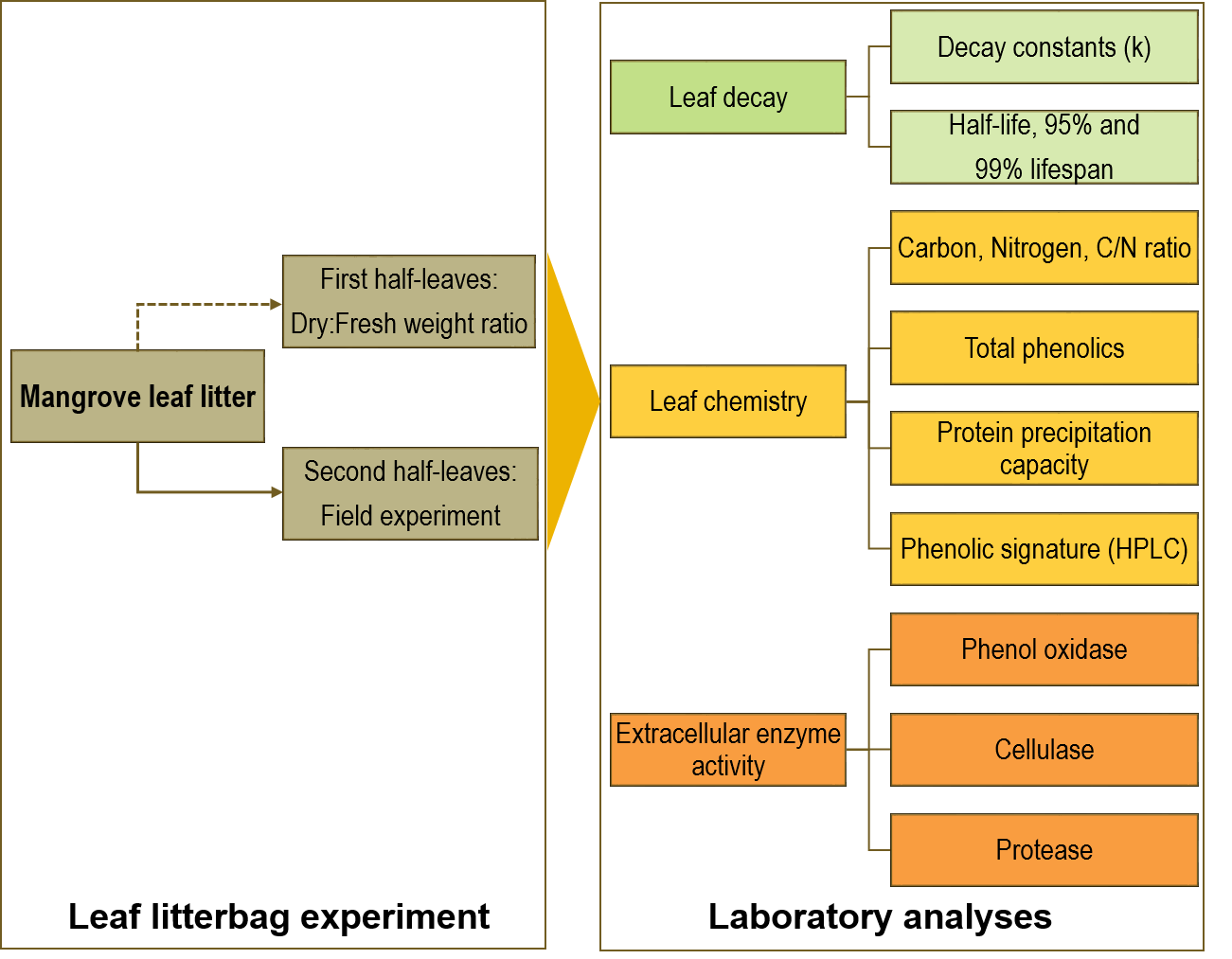 |
| --- |
| **Figure S1.3** Summary of field- and laboratory works conducted in this study. The measurement of leaf Dry:Fresh weight ratio and the analysis of leaf decay and extracellular enzyme activity were performed in the National University of Malaysia (UKM), while leaf chemistry analyses was conducted in the Leibniz Institute for Marine Tropical Research (ZMT), Germany. The measurement of carbon and nitrogen contents was performed to the initial (Day 0) and final (Day 70) samples (n=112), HPLC analysis was only conducted to the initial samples (n=56) and the other analyses were conducted to all samples (n=224). |

**Supplementary Material 2.** Results of Repeated Measures Analysis of Variance (ANOVA) of interspecific and ontogenetic differences on Leaf Litter Decay, Leaf Chemistry and Extracellular Enzyme Activity

**Table S2.1** Repeated measures ANOVA of the effects of species and ontogeny differences on leaf litter percent leaf mass remaining upon microbial decay (species x maturity x time).

| Source of difference | df | Mean square | F-ratio | *p*-value |
| --- | --- | --- | --- | --- |
| Percent Mass Remaining |  |  |  |  |
| Species | 3 | 8.07 | 19.69 | < 0.0001 |
| Maturity | 1 | 0.08 | 0.191 | 0.663 |
| Time | 3 | 41.2 | 100.558 | < 0.0001 |
| Species*Maturity | 3 | 1.27 | 3.103 | 0.028 |
| Species*Time | 9 | 2.62 | 6.389 | < 0.0001 |
| Maturity*Time | 3 | 0.1 | 0.238 | 0.870 |
| Species*Maturity*Time | 9 | 0.38 | 0.922 | 0.508 |
| Error | 160 | 0.41 |  |  |

**Table S2.2** Repeated measures ANOVA of the effects of species and ontogeny differences on leaf litter total carbon content upon microbial decay (species x maturity x time).

| Source of difference | df | Mean square | F-ratio | *p*-value |
| --- | --- | --- | --- | --- |
| Total carbon |  |  |  |  |
| Species | 3 | 0.29 | 3.962 | 0.011 |
| Maturity | 1 | 0.022 | 0.295 | 0.589 |
| Time | 1 | 0.030 | 0.414 | 0.522 |
| Species*Maturity | 3 | 0.069 | 0.950 | 0.420 |
| Species*Time | 3 | 0.050 | 0.690 | 0.561 |
| Maturity*Time | 1 | 0.025 | 0.339 | 0.562 |
| Species*Maturity*Time | 3 | 0.213 | 2.918 | 0.039 |
| Error | 80 | 0.073 |  |  |

**Table S2.3** Repeated measures ANOVA of the effects of species and ontogeny differences on leaf litter total nitrogen content upon microbial decay (species x maturity x time).

| Source of difference | df | Mean square | F-ratio | *p*-value |
| --- | --- | --- | --- | --- |
| Total nitrogen |  |  |  |  |
| Species | 3 | 0.048 | 5.241 | 0.002 |
| Maturity | 1 | 0.010 | 1.109 | 0.295 |
| Time | 1 | 0.353 | 38.394 | < 0.0001 |
| Species*Maturity | 3 | 0.009 | 0.946 | 0.422 |
| Species*Time | 3 | 0.003 | 0.373 | 0.773 |
| Maturity*Time | 1 | 0.0001 | 0.011 | 0.918 |
| Species*Maturity*Time | 3 | 0.044 | 4.801 | 0.004 |
| Error | 80 | 0.009 |  |  |

**Table S2.4** Repeated measures ANOVA of the effects of species and ontogeny differences on leaf litter carbon-to-nitrogen ratio upon microbial decay (species x maturity x time).

| Source of difference | df | Mean square | F-ratio | *p*-value |
| --- | --- | --- | --- | --- |
| C:N ratio |  |  |  |  |
| Species | 3 | 7.39 | 19.535 | < 0.0001 |
| Maturity | 1 | 1.39 | 3.673 | 0.059 |
| Time | 1 | 33.6 | 88.814 | < 0.0001 |
| Species*Maturity | 3 | 1.15 | 3.035 | 0.034 |
| Species*Time | 3 | 2.3 | 6.071 | 0.001 |
| Maturity*Time | 1 | 0.92 | 2.426 | 0.123 |
| Species*Maturity*Time | 3 | 1.66 | 4.387 | 0.006 |
| Error | 80 | 0.38 |  |  |

**Table S2.5** Repeated measures ANOVA of the effects of species and ontogeny differences on leaf litter total phenolic content upon microbial decay (species x maturity x time).

| Source of difference | df | Mean square | F-ratio | *p*-value |
| --- | --- | --- | --- | --- |
| Total phenolic content |  |  |  |  |
| Species | 3 | 26.38 | 55.473 | < 0.0001 |
| Maturity | 1 | 8.7 | 18.298 | < 0.0001 |
| Time | 3 | 108.99 | 229.211 | < 0.0001 |
| Species*Maturity | 3 | 0.29 | 0.604 | 0.614 |
| Species*Time | 9 | 4.35 | 9.15 | < 0.0001 |
| Maturity*Time | 3 | 4.5 | 9.462 | < 0.0001 |
| Species*Maturity*Time | 9 | 0.27 | 0.574 | 0.817 |
| Error | 160 | 0.48 |  |  |

**Table S2.6** Repeated measures ANOVA of the effects of species and ontogeny differences on leaf litter protein precipitation capacity upon microbial decay (species x maturity x time).

| Source of difference | df | Mean square | F-ratio | *p*-value |
| --- | --- | --- | --- | --- |
| Protein precipitation capacity |  |  |  |  |
| Species | 3 | 0.096 | 9.718 | < 0.0001 |
| Maturity | 1 | 0.063 | 6.352 | 0.013 |
| Time | 3 | 0.382 | 38.655 | < 0.0001 |
| Species*Maturity | 3 | 0.030 | 3.068 | 0.030 |
| Species*Time | 9 | 0.071 | 7.225 | < 0.0001 |
| Maturity*Time | 3 | 0.023 | 2.319 | 0.078 |
| Species*Maturity*Time | 9 | 0.010 | 1.049 | 0.404 |
| Error | 160 | 0.010 |  |  |

**Table S2.7** Repeated measures ANOVA of the effects of species and ontogeny differences on leaf litter phenol oxidase activity upon microbial decay (species x maturity x time).

| Source of difference | df | Mean square | F-ratio | *p*-value |
| --- | --- | --- | --- | --- |
| Phenol oxidase activity |  |  |  |  |
| Species | 3 | 273.6 | 44.768 | < 0.0001 |
| Maturity | 1 | 0.05 | 0.008 | 0.927 |
| Time | 3 | 204.93 | 33.532 | < 0.0001 |
| Species*Maturity | 3 | 100.05 | 16.37 | < 0.0001 |
| Species*Time | 9 | 45.84 | 7.501 | < 0.0001 |
| Maturity*Time | 3 | 1.24 | 0.203 | 0.894 |
| Species*Maturity*Time | 9 | 29.78 | 4.873 | < 0.0001 |
| Error | 160 | 6.11 |  |  |

**Table S2.8** Repeated measures ANOVA of the effects of species and ontogeny differences on leaf litter protease activity upon microbial decay (species x maturity x time).

| Source of difference | df | Mean square | F-ratio | *p*-value |
| --- | --- | --- | --- | --- |
| Protease activity |  |  |  |  |
| Species | 3 | 2.649 | 7.53 | < 0.0001 |
| Maturity | 1 | 2.85 | 8.099 | 0.005 |
| Time | 3 | 4.566 | 12.978 | < 0.0001 |
| Species*Maturity | 3 | 0.921 | 2.618 | 0.053 |
| Species*Time | 9 | 2.275 | 6.466 | < 0.0001 |
| Maturity*Time | 3 | 0.326 | 0.926 | 0.429 |
| Species*Maturity*Time | 9 | 0.61 | 1.734 | 0.085 |
| Error | 160 | 0.352 |  |  |

**Table S2.9** Repeated measures ANOVA of the effects of species and ontogeny differences on leaf litter cellulase activity upon microbial decay (species x maturity x time).

| Source of difference | df | Mean square | F-ratio | *p*-value |
| --- | --- | --- | --- | --- |
| Cellulase activity |  |  |  |  |
| Species | 3 | 7.711 | 9.902 | < 0.0001 |
| Maturity | 1 | 2.111 | 2.71 | 0.102 |
| Time | 3 | 14.63 | 18.786 | < 0.0001 |
| Species*Maturity | 3 | 4.308 | 5.531 | 0.001 |
| Species*Time | 9 | 3.93 | 5.047 | < 0.0001 |
| Maturity*Time | 3 | 2.749 | 3.53 | 0.0163 |
| Species*Maturity*Time | 9 | 0.966 | 1.241 | 0.274 |
| Error | 160 | 0.779 |  |  |

**Supplementary Material 3.** Results of High-Performance Liquid Chromatography (HPLC) presented as relative peak area of phenolic composition of initial mangrove leaf litter (Week 0)


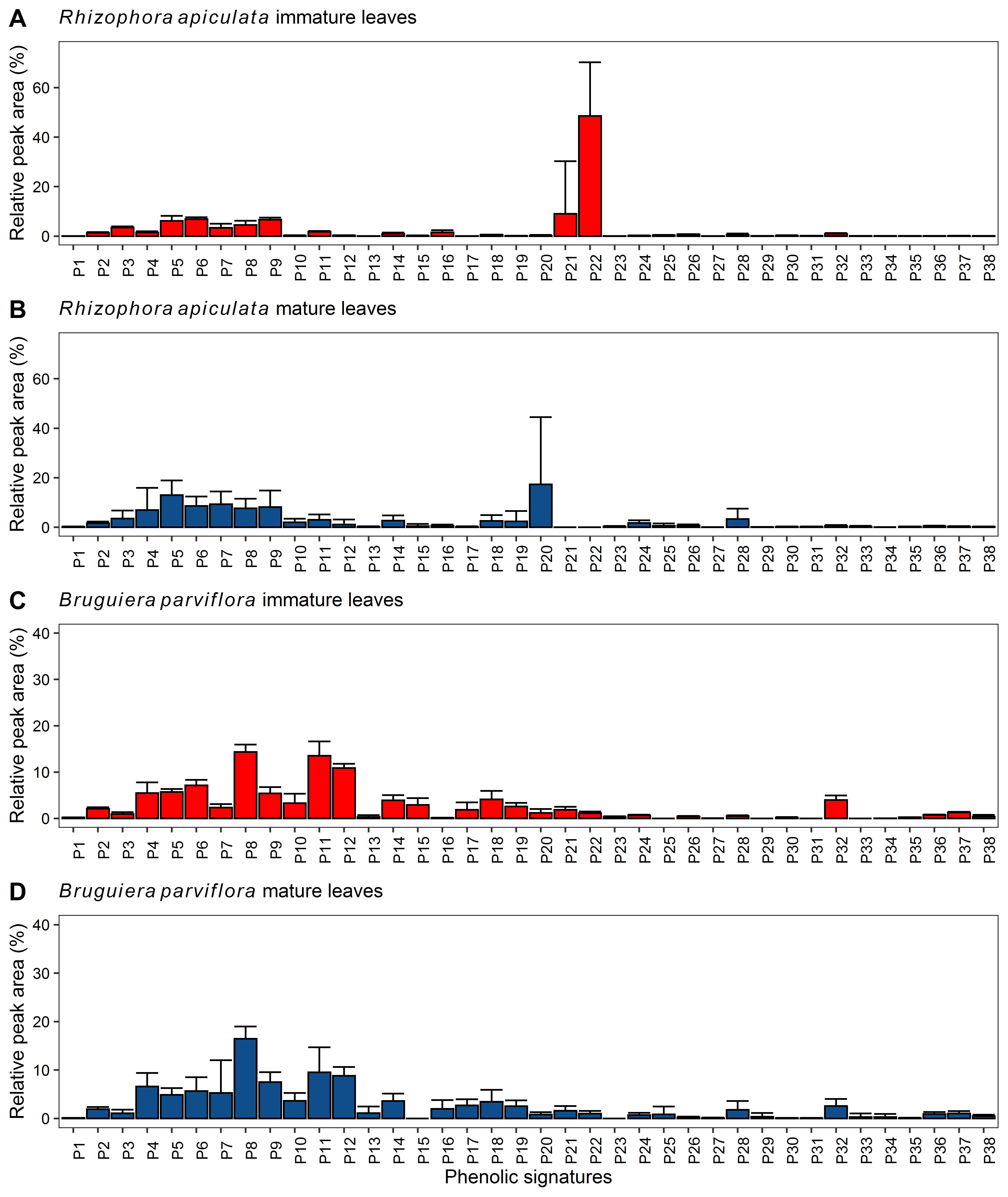


**Figure S3.1** Initial leaf litter phenolic composition of woody plants (a-b) *Rhizophora apiculata* and (c-d) *Bruguiera parviflora* in different ontogenetic stages (immature and mature stages). The error bars indicate the standard deviation of the replicates (n = 7). Tannic acid signatures are represented in peak number 6, 11, 22, and 28.


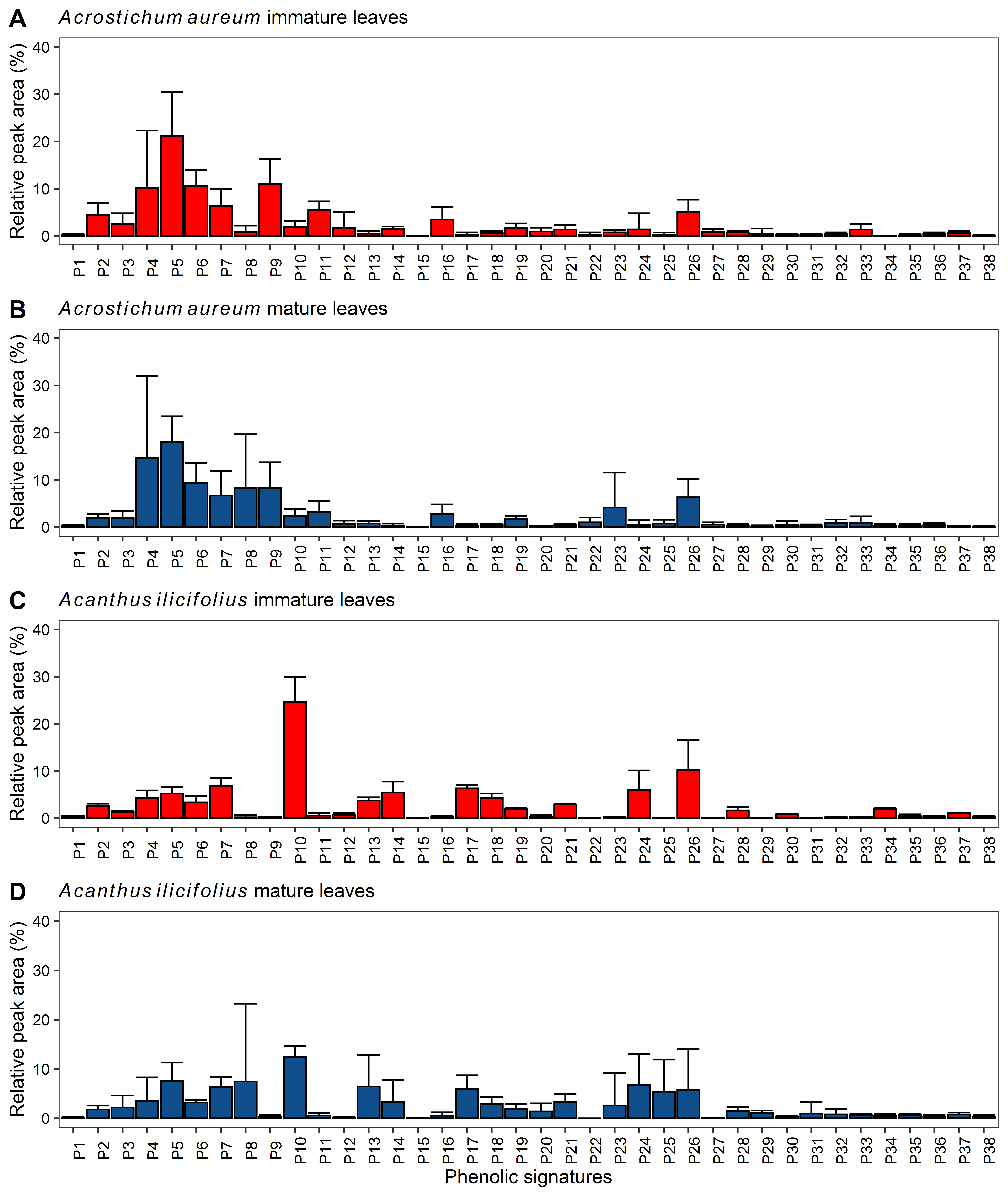


**Figure S3.2** Initial leaf litter phenolic composition of herbaceous plants (a-b) *Acrostichum aureum* and (c-d) *Acanthus ilicifolius* in different ontogenetic stages (immature and mature stages). The error bars indicate the standard deviation of the replicates (n = 7). Tannic acid signatures are represented in peak number 6, 11, 22, and 28.

Table S3.1 Similarity Percentage (SIMPER) analysis of phenolic signature peaks

| Peak | Av. dissim. | Contrib. % | Cumul. % | Mean  RA.IM | Mean RA.M | Mean BP.IM | Mean BP.M | Mean AA.IM | Mean AA.M | Mean  AI.IM | Mean  AI.M |
| --- | --- | --- | --- | --- | --- | --- | --- | --- | --- | --- | --- |
| P22 | 6.21 | 10.56 | 10.56 | 48.60 | 0.00 | 1.13 | 1.01 | 0.32 | 1.00 | 0.00 | 0.00 |
| P8 | 4.61 | 7.83 | 18.39 | 4.50 | 7.68 | 14.30 | 16.40 | 0.79 | 8.32 | 0.19 | 7.46 |
| P10 | 4.23 | 7.19 | 25.58 | 0.18 | 1.98 | 3.29 | 3.64 | 1.95 | 2.31 | 24.70 | 12.50 |
| P5 | 4.09 | 6.95 | 32.53 | 6.18 | 13.00 | 5.71 | 4.87 | 21.10 | 18.00 | 5.24 | 7.58 |
| P4 | 3.77 | 6.40 | 38.93 | 1.44 | 6.96 | 5.48 | 6.61 | 10.20 | 14.70 | 4.33 | 3.48 |
| P9 | 3.01 | 5.12 | 44.05 | 6.67 | 8.14 | 5.40 | 7.51 | 10.90 | 8.32 | 0.25 | 0.34 |
| P11 | 2.82 | 4.79 | 48.84 | 1.73 | 3.01 | 13.50 | 9.54 | 5.57 | 3.19 | 0.57 | 0.59 |
| P26 | 2.59 | 4.41 | 53.24 | 0.70 | 0.68 | 0.41 | 0.23 | 5.10 | 6.30 | 10.20 | 5.76 |
| P20 | 2.39 | 4.07 | 57.31 | 0.12 | 17.30 | 1.20 | 0.82 | 0.94 | 0.16 | 0.33 | 1.42 |
| P7 | 2.33 | 3.96 | 61.28 | 3.35 | 9.29 | 2.34 | 5.26 | 6.34 | 6.68 | 6.90 | 6.36 |
| P12 | 2.25 | 3.83 | 65.11 | 0.28 | 1.08 | 10.90 | 8.82 | 1.69 | 0.67 | 0.69 | 0.14 |
| P6 | 2.14 | 3.63 | 68.74 | 6.83 | 8.63 | 7.14 | 5.66 | 10.60 | 9.28 | 3.38 | 3.18 |
| P21 | 1.73 | 2.94 | 71.68 | 9.00 | 0.00 | 1.87 | 1.59 | 1.34 | 0.50 | 2.92 | 3.33 |
| P24 | 1.68 | 2.85 | 74.53 | 0.13 | 1.71 | 0.66 | 0.73 | 1.38 | 0.50 | 6.05 | 6.78 |
| P17 | 1.54 | 2.61 | 77.15 | 0.02 | 0.13 | 1.87 | 2.70 | 0.27 | 0.28 | 6.35 | 5.93 |
| P14 | 1.41 | 2.40 | 79.54 | 0.98 | 2.71 | 3.89 | 3.60 | 1.46 | 0.32 | 5.46 | 3.27 |
| P13 | 1.28 | 2.17 | 81.71 | 0.02 | 0.14 | 0.29 | 1.09 | 0.47 | 0.76 | 3.75 | 6.46 |
| P18 | 1.22 | 2.08 | 83.79 | 0.45 | 2.55 | 4.12 | 3.45 | 0.64 | 0.44 | 4.34 | 2.89 |
| P3 | 0.98 | 1.67 | 85.46 | 3.48 | 3.54 | 0.93 | 1.08 | 2.56 | 1.86 | 1.33 | 2.19 |
| P23 | 0.97 | 1.64 | 87.10 | 0.01 | 0.18 | 0.28 | 0.00 | 0.75 | 4.13 | 0.18 | 2.60 |
| P16 | 0.91 | 1.55 | 88.65 | 1.53 | 0.57 | 0.11 | 2.00 | 3.48 | 2.81 | 0.38 | 0.55 |
| P25 | 0.89 | 1.52 | 90.16 | 0.33 | 0.57 | 0.00 | 0.84 | 0.27 | 0.72 | 0.00 | 5.40 |
| P19 | 0.83 | 1.41 | 91.57 | 0.11 | 2.35 | 2.57 | 2.52 | 1.63 | 1.74 | 1.96 | 1.86 |
| P32 | 0.79 | 1.35 | 92.92 | 0.90 | 0.50 | 3.98 | 2.56 | 0.37 | 0.84 | 0.19 | 0.81 |
| P28 | 0.73 | 1.24 | 94.16 | 0.55 | 3.30 | 0.52 | 1.78 | 0.72 | 0.31 | 1.65 | 1.47 |
| P2 | 0.63 | 1.06 | 95.22 | 1.33 | 1.63 | 2.11 | 1.93 | 4.49 | 1.86 | 2.68 | 1.77 |
| P15 | 0.41 | 0.69 | 95.92 | 0.08 | 0.45 | 2.92 | 0.00 | 0.00 | 0.00 | 0.00 | 0.02 |
| P33 | 0.36 | 0.60 | 96.52 | 0.05 | 0.22 | 0.00 | 0.29 | 1.35 | 0.93 | 0.31 | 0.63 |
| P34 | 0.32 | 0.54 | 97.06 | 0.06 | 0.03 | 0.02 | 0.30 | 0.01 | 0.26 | 1.95 | 0.46 |
| P37 | 0.31 | 0.53 | 97.60 | 0.04 | 0.29 | 1.30 | 1.05 | 0.61 | 0.16 | 1.07 | 0.79 |
| P29 | 0.24 | 0.40 | 98.00 | 0.03 | 0.05 | 0.00 | 0.37 | 0.44 | 0.14 | 0.00 | 1.12 |
| P36 | 0.21 | 0.36 | 98.37 | 0.04 | 0.38 | 0.74 | 0.90 | 0.41 | 0.44 | 0.41 | 0.39 |
| P30 | 0.20 | 0.34 | 98.70 | 0.16 | 0.14 | 0.14 | 0.06 | 0.16 | 0.49 | 0.82 | 0.30 |
| P31 | 0.19 | 0.33 | 99.03 | 0.08 | 0.17 | 0.00 | 0.04 | 0.29 | 0.34 | 0.07 | 0.95 |
| P27 | 0.18 | 0.30 | 99.33 | 0.01 | 0.02 | 0.01 | 0.07 | 0.86 | 0.55 | 0.09 | 0.10 |
| P35 | 0.16 | 0.27 | 99.60 | 0.02 | 0.22 | 0.23 | 0.04 | 0.11 | 0.32 | 0.46 | 0.62 |
| P38 | 0.15 | 0.25 | 99.85 | 0.03 | 0.21 | 0.41 | 0.50 | 0.07 | 0.14 | 0.37 | 0.39 |
| P1 | 0.09 | 0.15 | 100.00 | 0.05 | 0.17 | 0.19 | 0.11 | 0.31 | 0.27 | 0.42 | 0.14 |

**Note:** Av. dissim. = average dissimilarity; Contrib. % = percentage of contribution; Cumul. % = cumulative percentage of contribution; RA.IM = immature *Rhizophora apiculata*; RA.M = mature *R. apiculata*; BP.IM = immature *Bruguiera parviflora*; BP.M = mature *B. parviflora*; AA.IM =immature *Acrostichum aureum*; AA.M = mature *A. aureum*; AI.IM = immature *Acanthus ilicifolius*; AI.M = mature *A. ilicifolius*.
